# Simiate and the focal adhesion kinase FAK1 cooperate in the regulation of dendritogenesis

**DOI:** 10.1038/s41598-022-14460-y

**Published:** 2022-07-04

**Authors:** Ramya Rama, Kristin Derlig, Nina Vießmann, Roman Gossmann, Fabian Oriold, Andreas Gießl, Johann Helmut Brandstätter, Ralf Enz, Regina Dahlhaus

**Affiliations:** 1 Institute for Biochemistry, Emil-Fischer Centre, Friedrich-Alexander-Universität Erlangen-Nürnberg, 91054 Erlangen, Germany; 2 Department of Biology, Animal Physiology, Friedrich-Alexander-Universität Erlangen-Nürnberg, 91058 Erlangen, Germany; 3 Institute of Neuroscience and Medicine, INM-2 and INM-10, Research Centre Jülich, 52425 Jülich, Germany; 4 Department of Psychiatry, Psychotherapy and Psychosomatics, RWTH Aachen, 52074 Aachen, Germany; 5 Department of Ophthalmology, University Hospital Erlangen, Schwabachanlage 6, 91054 Erlangen, Germany; 6 Research Division for Neurodegenerative Diseases, Faculty of Medicine/Dentistry, Danube Private University, Steiner Landstrasse 124, 3500 Krems-Stein, Austria

**Keywords:** Cytoskeleton, Neuronal development, Biochemistry

## Abstract

Despite the crucial importance of dendritogenesis for the correct functioning of neurons, the molecular mechanisms underlying neuronal arborisation are still not well understood. Current models suggest that distinct parts and phases of dendritic development are regulated by the expression of distinct transcription factors, that are able to target the cytoskeleton. Two proteins recently implicated in dendritogenesis are the Focal Adhesion Kinase FAK1 and the Actin-binding protein Simiate. Using heterologous expression systems as well as mouse brain extracts in combination with coprecipitation assays, we show that Simiate is able to associate with FAK1. Differential centrifugation experiments further revealed the interaction to be present in cytosolic as well as nuclear fractions. Inside the nucleus though, Simiate preferentially binds to a FAK1 isoform of 80 kDa, which has previously been shown to regulate transcription factor activity. Investigating the function of both proteins in primary hippocampal cultures, we further found that FAK1 and Simiate have distinct roles in dendritogenesis: While FAK1 increases dendrite length and number, Simiate preferentially enhances growth and branching. However, if being confined to the nucleus, Simiate selectively triggers primary dendrite formation, enhancing transcription activity at the same time. Since the effect on primary dendrites is specifically re-normalized by a co-expression of FAK1 and Simiate in the nucleus, the data implies that the two proteins interact to counterbalance each other in order to control dendrite formation. Looking at the role of the cytosolic interaction of FAK1 and Simiate, we found that neurotrophin induced dendritogenesis causes a striking colocalisation of FAK1 and Simiate in dendritic growth cones, which is not present otherwise, thus suggesting that the cytosolic interaction stimulates growth cone mediated dendritogenesis in response to certain external signals. Taken together, the data show that FAK1 and Simiate exert several and distinct actions during the different phases of dendritogenesis and that these actions are related to their subcellular localisation and their interaction.

## Introduction

Understanding the molecular mechanisms that route the delicate development of dendritic trees has proven a fundamental challenge in neuroscience. Governed by a variety of extrinsic signals such as adhesion molecules, growth factors and neuronal activity as well as intrinsic factors like chromatin remodelling proteins, Histone modifying enzymes and transcription factors (reviewed in^1–3^), dendritogenesis was found to be chiefly regulated by changes in the expression of transcription factors. Indeed, even shifts in a single transcription factor have been found sufficient to significantly alter the dendritic arborisation as well as the projection patterns of a neuron (reviewed in^1,2^).

These investigations have given rise to a model where different transcription factors are detailed to distinct facets and phases of dendritogenesis (reviewed in^1^), although both, the proteins involved as well as the molecular mechanisms underlying the effects have mostly remained elusive. Remarkably though, components of the cytoskeleton such as Actin, Gelsolin or microtubule binding proteins have been shown to be targets of transcription factors in the context of dendritogenesis^4–11^, thus suggesting that dendritic arborisation is driven by diverse connections of transcription factors on the one side and the cytoskeleton on the other.

In line with this model, we have recently shown that Simiate, a protein indicated to function in transcription and/or splicing processes^12–14^, directly associates with Actin, and increases dendritic complexity upon enhanced expression^13^. Moreover, while absent from axons and synapses, endogenous Simiate alters its colocalisation with Actin significantly during dendritogenesis, in particular in dendritic growth cones. Further studies suggested that an interaction with the Focal Adhesion Kinase FAK1 (alias: Protein Tyrosine Kinase 2) might have a role in this context^13^.

FAK1 is a 120 kDa scaffolding protein, which is prominent at focal adhesion sites of many cell types and functions in cytoskeleton organisation by directly enhancing Wasp and Arp2/3-mediated Actin polymerization, for example during cell migration (reviewed in^15^). In the mammalian brain, where FAK1 was found to serve in neuronal migration and arborisation as well as in synaptic plasticity, multiple isoforms have been detected, which are generated by both, alternative splicing and proteolytic cleavage (reviewed in^16^). The specific cellular roles of these isoforms are widely unknown though.

Interestingly, recent research revealed that FAK1 shuttles into the nucleus, where it was found to function in chromatin remodelling, probably by associating with the DNA binding protein MBD2, and to mediate the degradation of a transcription factor, GATA4^17–19^. While studies in endothelial cells illustrated FAK1 and GATA4 to serve in cell proliferation, the neuronal functions of GATA4 are unknown^20^. However, FAK1 induced Chromatin remodelling by MBD2 was shown to serve in the differentiation of muscle cells^17,21^, suggesting that nuclear FAK1 might have a role in gene expression regulation during neuronal development.

Focusing on the function of Simiate and FAK1 in dendritogenesis, we show that nuclear Simiate specifically enhances primary dendrite development, while somatic Simiate serves to stimulate dendrite growth and branching. Demonstrating that Simiate binds to FAK1 and the 80 kDa Calpain cleavage fragment "FAK80"^22,23^ in a developmentally regulated manner, we further show that a balanced nuclear expression of FAK1 and Simiate serves to control primary dendrite formation by regulating gene expression.

## Methods

### Biochemistry

#### Production of recombinant proteins

The manufacturing of GFP-, Glutathione-sepharose-tag- (GST-) and FLAG- (DYKDDDDK-) tagged Simiate was published previously^12^. A clone containing FAK1 in the pGZ21dxZ vector^24^ was purchased from Addgene and expressed in HEK293 cells to obtain Green-Fluorescent-Protein- (GFP-) tagged FAK1, while expression in primary hippocampal neurons served to study the function of FAK1 in dendritogenesis. By contrast, GFP labelled NLS-Simiate was generated by PCR using primers with nuclear localisation signals (forward: ATGGATCCGAAGAAAAAACGTAAA-ATGGAAGAGCTCCGCTGC and reverse: TCAATCCAGTTTCACGCGTTTCGCCGCCG-GGGGCGTGGTGGCTGTGG) and subsequent cloning into pDest732 (Gateway, *Life Technologies*), thus resulting in the protein GFP-NLS-Simiate-NLS ("GFP-NLS-Simiate") upon expression. To perform coprecipitation assays, GST- fusion proteins were affinity purified from E. coli BL21 Rosetta (*Novagen*) cells as outlined in the manufacturer's instructions (*GE Healthcare*) using a French press (*Thermo Electron*) for cell lysis.

#### Preparation of nuclear proteins

In order to extract proteins from cell nuclei in a condition suitable for coprecipitation assays, a preparation procedure employing sucrose gradient centrifugation and ultra sound was established. Per sample, either 3–5 adult mouse brains or two 10 cm-dishes transfected HEK293 cells were homogenised in app. 3–5 ml Hepes-buffer (10 mM HEPES, pH 7.5; 1 mM EGTA; 0.1 mM MgCl_2_; 1% Triton; 150 mM NaCl) using either 25–30 pestle strokes with a teflon Dounce homogenizer (mouse brains) or up and down pipetting with a small tip (HEK293 cells). The homogenate obtained was centrifuged for 15 min at 170rcf and 4 °C, and the resultant pellet was resuspended in app. 1 ml buffer 1 (0.32 M Sucrose, 20 mM Hepes, pH 7.4, 2 mM MgCl_2_, 43 mM β-Mercaptoethanol, 1 × protease inhibitor (*Roche*)). After another centrifugation at 500rcf and 4 °C for 15 min, the pellet was again resuspended in buffer 1, and buffer 2 (2.4 M Sucrose and 2 mM MgCl_2_) was added in a ratio of 1:2, before the sample was subjected to ultracentrifugation for 1 h at 70000rcf and 4 °C (or 45 min. at 90000rcf). Following resuspension of the new pellet in buffer 1 as well as addition of buffer 2 (ratio: 1:2), the solution was laid on buffer 3 (2.15 M Sucrose and 2 mM MgCl_2_) and subjected to another ultracentrifugation. After resuspending the resultant pellet in 200 µl Ripa buffer (50 mM Tris–HCl, pH 7.5, 500 mM NaCl, 1%NP40, 0.5% Sodiumdesoxycholate, 1 × protease inhibitor without EDTA), the sample was incubated at 4 °C for 30 min, before 30 ultra sound strikes (50 Watt) with 30 s breaks were applied. Following a last centrifugation (500rcf, 4 °C, 5 min), extracted proteins were diluted with Ripa buffer to achieve the desired conditions.

#### Coprecipitations

A detailed description of non-covalent coprecipitation assays has been reported recently^13^. Briefly, GFP recombinant proteins expressing HEK293 cells were lysed and the cytosolic fraction was incubated over night at 4 °C with Glutathione sepharose beads *(GE Healthcare)* carrying either 50 µg GST-Simiate or GST solo. Following three washing steps, adsorbed proteins were eluted at 60 °C for 20 min in SDS-buffer (16% SDS; 40% Glycerine; 250 mM Tris–HCl, pH 6,8; 4% *beta*-Mercaptoethanol; Bromophenol blue) and subjected to SDS-PAGE and western blotting. To perform coprecipitations with FAK1 proteins, Ripa-buffer (50 mM Tris–HCl, pH 7.5; 0.5–1% NP-40, 0.5% Sodiumdesoxycholate, 1 × protease inhibitor with 100-300 mM NaCl and 0.5–1% Triton) was used.

### Histology

#### Cell culture and fluorescence experiments

For details of the cultivation and immunhistochemistry of E17-E18 rat primary hippocampal neurons as well as immunofluorescence labelling of brain slices, please refer to^12^ and ^25^. To perform Sholl analyses, primary hippocampal neurons were transfected at day in vitro (div) 7 using Lipofectamine 2000 or 3000 (both *Life technologies*) and PFA-fixated 24 h later.

#### Treatment with Nerve growth factor

Following the procedure established by Monje et al.^26^, 25 ng/ml nerve growth factor (NGF, *Life technologies*) were applied to the medium of div 2 primary hippocampal neurons. After over night incubation at regular conditions, cells were processed for analyses as usual^12,25,27^.

#### Antibodies and dyes

Alexa-antibodies (*Life technologies*; IHC 1:500–1:1000), Alexa Fluor Acid 647 (click-it labelling, *Life Technologies*), Click-it RNA imaging Kit (*Life Technologies*), 4′,6-Diamidin-2-phenylindol (DAPI, Chromatin staining, *Sigma-Aldrich*; IHC 1:50,000).), DNase Alexa488 (G-Actin and DNA labelling; *Life Technologies*), FAK1 (mouse, 10H7E9, *Abcam* or *Biomol*; WB: 1:500, IHC: 1:200), GFP (mouse; *Covance*; WB 1:2000), gtαmsCy5 (*Abcam*; IHC 1:250), Histon4 Lysine20 di-tri Methyl (H4K20me2/3, mouse, *Abcam;* IHC 1:200), heterogeneous nuclear Ribonucleoproteins (hnRNP A2B1 mouse, *Abcam*; IHC 1:200), Horse Radish Peroxidase antibodies (GE Healthcare; WB 1:2500), Microtubule associated Protein 2 (MAP2, chicken, *Abcam*; IHC: 1:2500), active RNA Polymerase 2 (RNAP2, rabbit, *Abcam*; IHC 1:200), Simiate (rabbit,^12^; WB: 1:2000; IHC: 1:200).

#### Microscopy

All imaging was implemented using either a laser scanning microscope (LSM 710, *Zeiss,* and SP5 II, *Leica*), or, to perform Sholl analyses, a conventional fluorescence microscope (Axiophot, *Zeiss*).

### Bioinformatics and statistics

#### Morphometry

In order to analyse the volume of Simiate and FAK1 present in different cellular compartments, confocally imaged z-stacks were employed to reconstruct protein distributions three-dimensionally using Imaris 8 and 9 (*Bitplane,* https://imaris.oxinst.com/) software^28^. The precise localisations of FAK1 and Simiate were investigated using ImageJ 1.50—1.52 (*NIH,* https://imagej.nih.gov/ij/) to quantify corresponding signal intensities on microscopic pictures. Furthermore, Fiji 2.0.0–2.1.0 (NIH, https://fiji.sc/) were employed to produce z-projections and colocalisation images from files generated by Laser scanning microscopy. To investigate the arborisation of neurons, Sholl analyses were implemented with 15 concentrical cycles and a cycle distance of 10 µm (cp.^13^) using Fiji with the corresponding ImageJ plug-in. In addition, primary, secondary and tertiary dendrites were tracked and measured with NeuronJ in Fiji^29^. Neurons showing protein aggregation due to high construct expression, increased vacuole formation or signs of loss of morphological integrity were excluded from any analyses.

#### Statistical testing

All statistical analyses were performed either in Prism 5 and 9 (*GraphPad Software Inc*., https://www.graphpad.com/scientific-software/prism/) or Excel 16 (*Microsoft Corp*., https://www.microsoft.com/de-de/microsoft-365/excel) and Calc (Libreoffice 6, https://www.libreoffice.org/discover/calc/) as written on before^12^. Briefly, comparisons were implemented by applying t-testing for single and analysis of variance (ANOVA) along with posthoc analyses for multiple computations and Barlett's test for variance. All results are displayed as either significant (*p* < 0.05, "*") or not (*p* > 0.05). The test details and results are summarized by Supplemental Table 1. In order to focus the presentation of corresponding data, some results are displayed as average of the % deviation from the mean value of the corresponding control as indicated in the corresponding figure legends. All error bars show the 95% confidence interval.

### Animal care

All animals employed in this study were kept at the rodent facility of the Institute for Biochemistry of the University Erlangen-Nürnberg in strict accordance with the guidelines of the Federation of European Laboratory Animal Science Associations and the recommendations of the European Convention for the Protection of Vertebrate Animals used for Experimental and Other Scientific Purposes of the Council of Europe (cp^12^). Protocols were approved by the Animal Care Committee of the University of Erlangen-Nuremberg (Permit number: TS-8/12 Biochemie) and animal welfare conventions are controlled regularly by the city of Erlangen (cp^12^). The experimentation is in compliance with ARRIVE guidelines. For further details on animal breeding, please see^12^.

## Results

### Simiate and focal adhesion kinase 1 associate

Simiate is an Actin-binding protein, which is present in dendrites as well as in nuclei. In the latter, it is localised to small compartments called nuclear speckles, which serve to organise the transcription- and splicing machineries (reviewed in^30^). Though Simiate contains no known nuclear localisation signal, being only 22 kDa in size, it is tiny enough to enter the nucleus based on diffusion.

Previous experiments illustrated that enhanced Simiate expression is sufficient to significantly increase the complexity of dendritic trees in primary hippocampal neurons^13^. Since Actin dynamics, focal adhesions and cellular morphology are intimately connected, we speculated that FAK1 might have a role in Simiate mediated dendritogenesis. Remarkably though, when studying the colocalisation of both proteins in neuronal as well as HEK293 cells, we observed a significant overlap of FAK1 and Simiate in nuclear speckles^13^. These findings made us curious about an association of the two proteins in the nucleus, and the role of their interaction in dendritogenesis.

**Figure 1:**
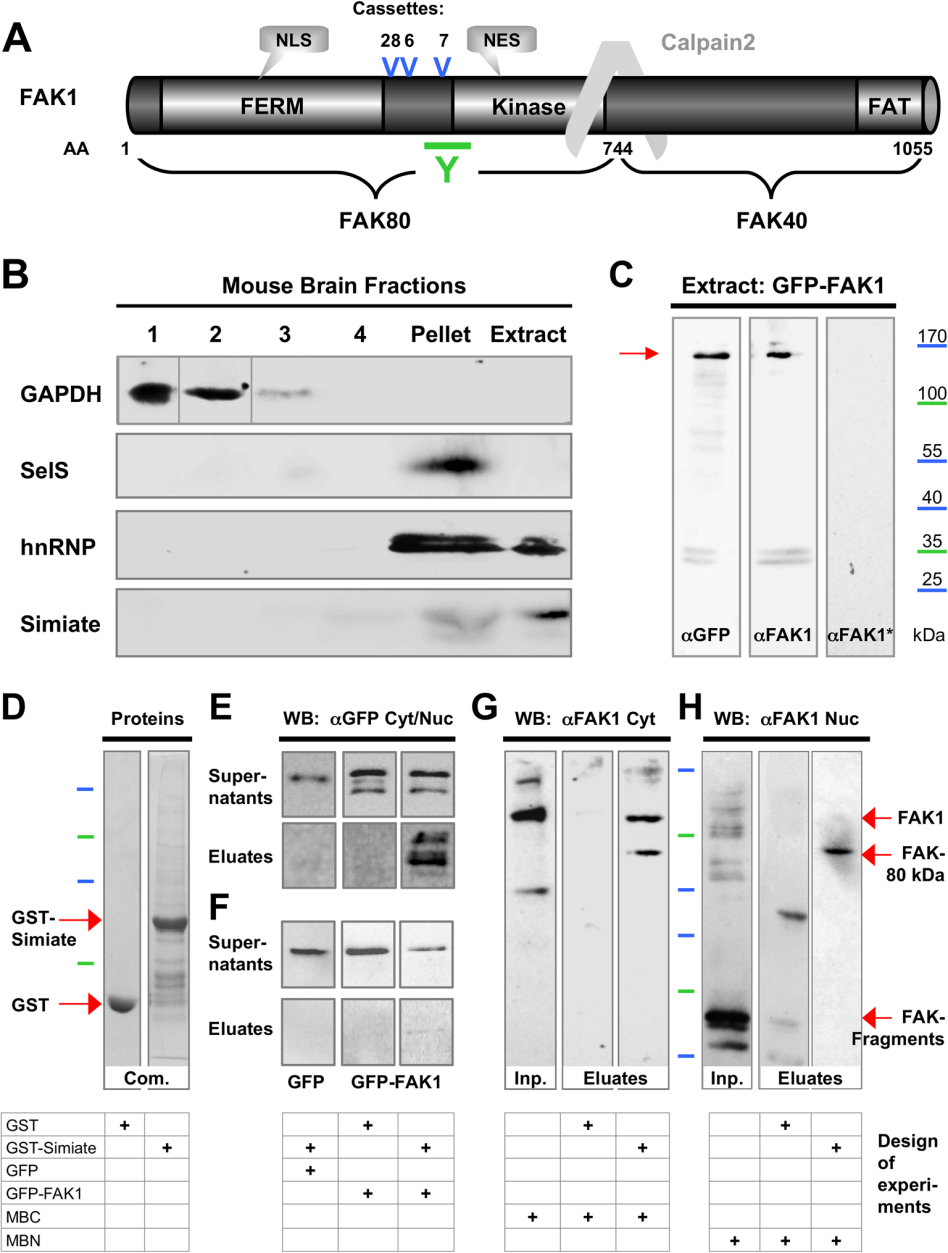
FAK1 and Simiate associate. (**A**) The picture shows a schematic representation of FAK1 and its isoforms. Due to alternative splicing (casettes 28, 6 and 7) and proteolytic cleavage by Calpain2, three groups can be detected: Full-length isoforms (118–122 kDa), FERM domain isoforms (45–48 kDa) and cleavage products (80 and 40 kDa). While FAK80, FERM domain or full-length proteins are recognised by our FAK1-antibody (green Y), FAK40 fragments cannot be detected. AA: Amino Acids. FERM: Band Four-point-one, Ezrin, Radixin and Moesin common domain. NES: Nuclear Export Signal. NLS: Nuclear Localisation Signal. FAT: Focal Adhesion Targeting. Blue V: Location of optional protein cassettes, which are present in full-length as well as FERM domain isoforms and contain 28, 6 or 7 additional amino acids. Y: Antibody binding site. (**B**) Nuclear fractioning of adult mouse brains. 1—Mouse brain homogenate, 2—Mouse brain cytosol, 3—Pellet 500rcf, 4—Supernatant 70000rcf, Pellet—final pellet containing nuclear proteins not extracted, Extract—proteins successfully extracted from cell nuclei. Due to a different gel loading order, lanes 1 and 2 of the first blot ("GADPH") were swapped after imaging to fit the figure. (**C**) Nuclear GFP-FAK1 is specifically detected by αFAK1. The Western blot stripes show extracts from nuclei of GFP-FAK1 expressing HEK293 cells stained αGFP, αFAK1 and αFAK1 preincubated with GFP-FAK1 (αFAK1*). (**D**–**H**) Coprecipitation assays with recombinant FAK1 (**E**,**F**) and endogenous FAK isoforms (**G**,**H**) demonstrate FAK1 to associate with Simiate. The specific experimental design is indicated by the table at the bottom of each experiment, while protein sizes are indicated in a colour coded manner (cp. **C**). (**D**) A Coomassie-staining visualises the proteins used as baits in the subsequent coprecipitation experiments. The experiments shown in (**E**) and (**F**) were performed with HEK293 cells, whereas the experiments shown in (**G**–**H**) were performed with mouse brain. (**E**) Cytosolic GFP-FAK1 binds to GST-Simiate. (**F**) Nuclear GFP-FAK1 also associates with Simiate. Left and middle panels in (**D**) and (**E**/**F**): GFP and GST controls showing the specificity of the assay. Please note that the presentation does not reflect actual protein seizes (GFP: 30 kDa vs. GFP-FAK1: 150 kDa, dotted line). (**G**) Simiate interacts with endogenous FAK1 and FAK80, a FAK1 isoform generated by Calpain2 cleavage (cp. **A**). H) In the nucleus, Simiate prefers FAK80. Both experiments (**G**,**H**) show a significant enrichment of FAK80 in the precipitate by Simiate, even from below detection levels (**G**), suggesting a strong association of both proteins. The FAK-fragments observed at the bottom of some blots shown in (**C**) and (**H**) are most likely caused by prolonged proteolytic activity during the preparation procedure, whereas bands > 120 kDa might represent additional isoforms generated by posttranslational modifications and/or alternative splicing as suggested by NCBI data bases, which predict further full length proteins in the range of 120–130 kDa based on alternative splicing. The nature of the additional band observed at app. 40 kDa in the GST-lane is unclear though, however, no low-weight bands are seen for GST-Simiate, suggesting an unspecific binding related to high concentrations of FAK-fragments and/or of solitary GST in the GST sample. All proteins were extracted from adult mouse brains. Com. = Commassie; Inp. = Input; MBC = Mouse Brain Cytosol; MBN = Mouse Brain Nucleoplasm.

To test the implied protein–protein interaction of FAK1 and Simiate, we decided to implement coprecipitation assays using immobilised GST-Simiate together with mouse brain extracts or GFP-FAK1 (Fig. 1 and Supplemental Figure for Fig. 1). Given that there are multiple FAK1 isoforms present in the mammalian brain (Fig. 1A; reviewed in^16^), the association could either involve specific FAK1 isoforms only and/or depend on the respective subcellular localisation. Since both, FAK1 and Simiate are present in the cytosol as well as in the nucleus, we developed a purification procedure for nuclear proteins based on gradient centrifugation and ultrasonic membrane disruption (Fig. 1B), which allows for an application of nuclear proteins in coprecipitation assays. An assessment of the fractions obtained revealed that the cytosolic protein GAPDH is abundant in the homogenate and the cytosol, but absent from the two nuclear fractions containing either extracted (Fig. 1B; Extract) or unextracted (Fig. 1B; Pellet) proteins. By contrast, Selenoprotein S (SelS), a protein located within the membrane of the endoplasmic reticulum^31,32^, is highly enriched in the fraction of unextracted proteins, while Simiate, a protein localised to nuclear speckles, is mainly seen in the fraction of extracted proteins. The procedure thus specifically purifies nuclear proteins, but not material from adjunct membrane systems or the cytosol.

Using this method, we next set out to prepare nuclear GFP-FAK1. Considering that site specific posttranslational modifications might impair antibody performance, we decided to first validate our FAK1 antibody on nuclear cell material. Therefore, we expressed GFP-FAK1 in HEK293 cells and subjected the extracted proteins to Western blotting (Fig. 1C). Stainings with anti-GFP, anti-FAK1 and anti-FAK1 preincubated with GFP-FAK1 (anti-FAK*) revealed that GFP-FAK1 is not only well present in nuclei, but also specifically detected by our FAK1 antibody.

In order to test the postulated protein–protein interaction of FAK1 and Simiate (Fig. 1D-F), we now applied nuclear as well as cytosolic GFP-FAK1 extracts from HEK293 cells in a GST-coprecipitation assay (Fig. 1E,F) using affinity purified GST-Simiate for bait and GST for control (Fig. 1D). While there is no association of GFP and GST-Simiate (Fig. 1E,F; left panels) or GFP-FAK1 and GST (Fig. 1E,F; middle panels), a profound copurification of GST-Simiate and GFP-FAK1 is seen for cytosolic GFP-FAK1 (Fig. 1E; right panel), and, to a lesser extend, for nuclear GFP-FAK1 (Fig. 1F; right panel), thus demonstrating that Simiate is able to specifically bind to both, cytosolic as well as nuclear FAK1.

Due to alternative splicing, the mammalian brain comprises several full-length FAK1 isoforms weighing app. 118–122 kDa, as well as several FERM domain variants of app. 45–48 kDa in weight (Fig. 1A, reviewed in^16^). In addition, a 80 kDa Calpain2 cleavage product composed of the FERM and the kinase domain is found in brain tissue ("FAK80",^18,22^). Hence, we tested a potential binding preference of Simiate for certain FAK1 isoforms by performing coprecipitation assays with affinity purified GST-Simiate and brain extracts from cytosolic as well as nuclear fractions obtained from adult mice (Fig. 1G,H). GST served for control. The experiments revealed Simiate to associate with several distinct FAK1 isoforms, whose molecular weights correspond to full length proteins and the FAK80 fragment (Fig. 1G,H). In the nucleus though, Simiate shows a significant preference for FAK80, resulting in a striking enrichment of this isoform in the precipitate (Fig. 1H). Indeed, the association of FAK80 and Simiate is seen even though FAK80 is almost below detection level in the inputs of both experiments (Fig. 1G,H, left lane versus right lane) and turned out to be stable under different buffer conditions, thus suggesting a robust interaction of Simiate and FAK80.

### The localisation of FAK1 and Simiate changes during neuronal development

**Figure 2:**
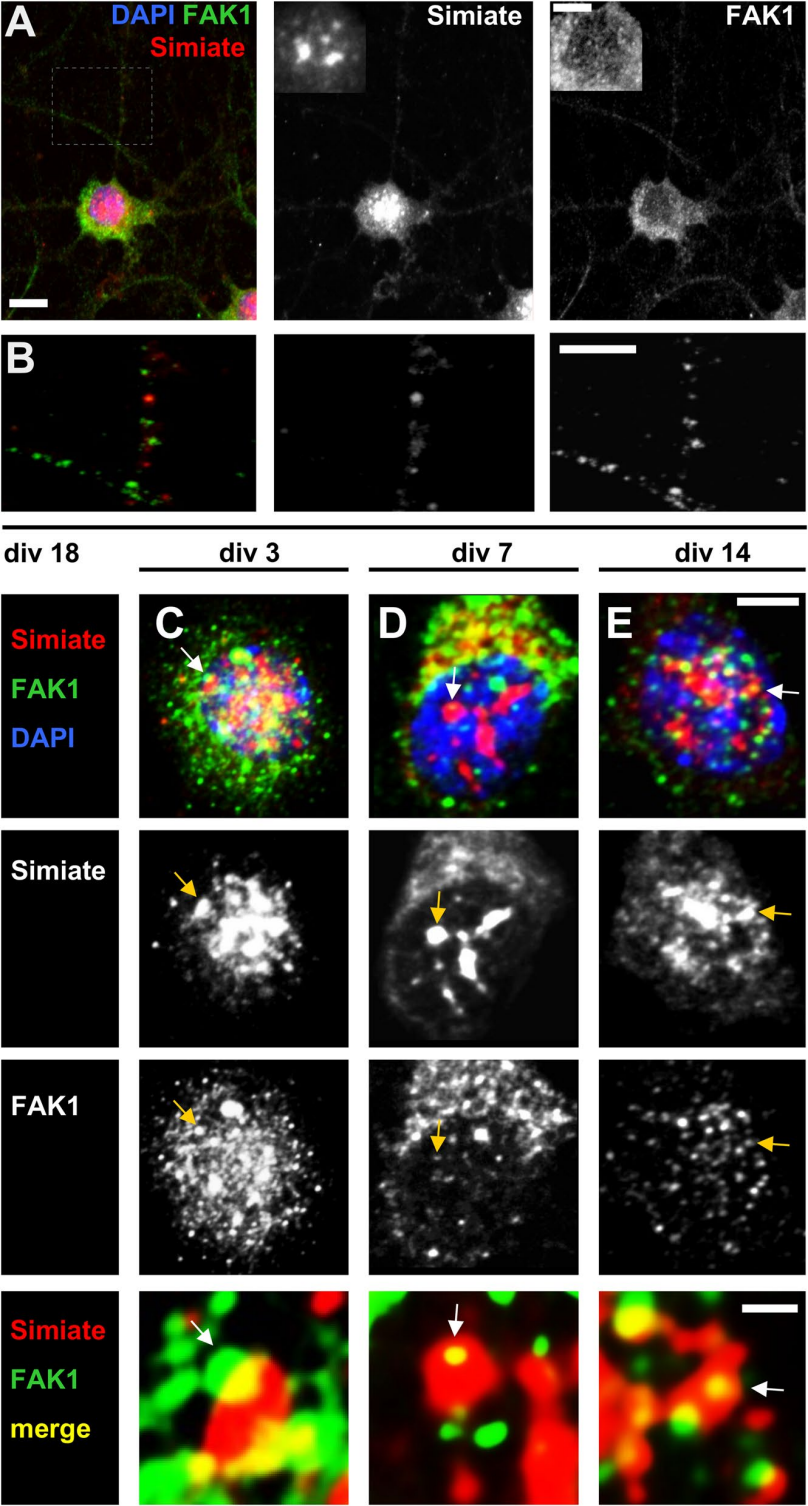
Simiate and FAK1 colocalise during neuronal development at changing extents. The pictures show z-projections of whole-cell z-stacks recorded confocally from cultured hippocampal neurons. (**A**) A div 18 neuron shows the characteristic expression patterns of FAK1 and Simiate: while FAK1 is mainly found in the soma and dendrites, Simiate is significantly enriched in the nucleus. There, it localises to nuclear speckles. Due to the high concentration in these compartments, dendritic Simiate is usually not seen unless the nuclear signal is already significantly oversaturated. A shorter exposure of the nuclear Simiate signal is shown in the insert. Scale bars: 10 µm and 5 µm in the insert. (**B**) A magnification of the boxed region in (**A**) illustrates the localisation of FAK1 and Simiate in dendrites. Scale bar: 5 µm. Please note that the region has been selected to include a dot, which is demonstrating a colocalisation of FAK1 and Simiate. (**C**–**E**) Simiate and FAK1 colocalising in developing neurons at div 3 (**A**), at div 7 (**B**) and at div 14 (**C**). Scale bars: 5 µm and 1 µm in the magnification. In the bottom panel, magnifications of the speckles indicated by arrows are shown. A colocalisation of Simiate and FAK1 is often seen in the outskirts of nuclear speckles (white/yellow arrows, cp. magnification in (**A**). The pictures are contrasted to visualise the colocalisation of both proteins (yellow). For a detailed quantification, please see the next figures.

When looking at the expression of FAK1 and Simiate in mature neurons (Fig. 2A,B), FAK1 is most abundant in somata and dendrites, whereas Simiate is significantly enriched in nuclear speckles. However, some Simiate is also present in dendrites (Fig. 2B) and likewise, some FAK1 is found in nuclei and nuclear speckles (Fig. 2A). A colocalisation outside the nucleus is rare though (Fig. 2B, green and red dots).

Considering the arbour-promoting function of FAK1 and Simiate in dendritogenesis^13,26^, their physical interaction (Fig. 1) and their colocalisation in nuclear speckles^13^, we wondered whether their localisation and thus their potential association might be involved in dendritogenesis. To address this question, we first performed colocalisation studies in differentiating hippocampal neurons (Fig. 2C–E).

At div 3, both proteins show a strong signal in the nucleus (Fig. 2C), but while the nuclear FAK1-signal diminishes during the following days (Fig. 2D,E), the signal of nuclear Simiate only shows a minor reduction around div7 before increasing again at div 14 (Fig. 2D,E). Looking at the colocalisation of nuclear FAK1 and Simiate, the experiments show striking alterations in the appearance of both proteins: while a profound colocalisation of endogenous FAK1 and Simiate is seen in nuclear speckles of div 3 neurons (Fig. 2C), their colocalisation almost disappears around div 7 (Fig. 2D) and comes back at about div 14 (Fig. 2E).

**Figure 3:**
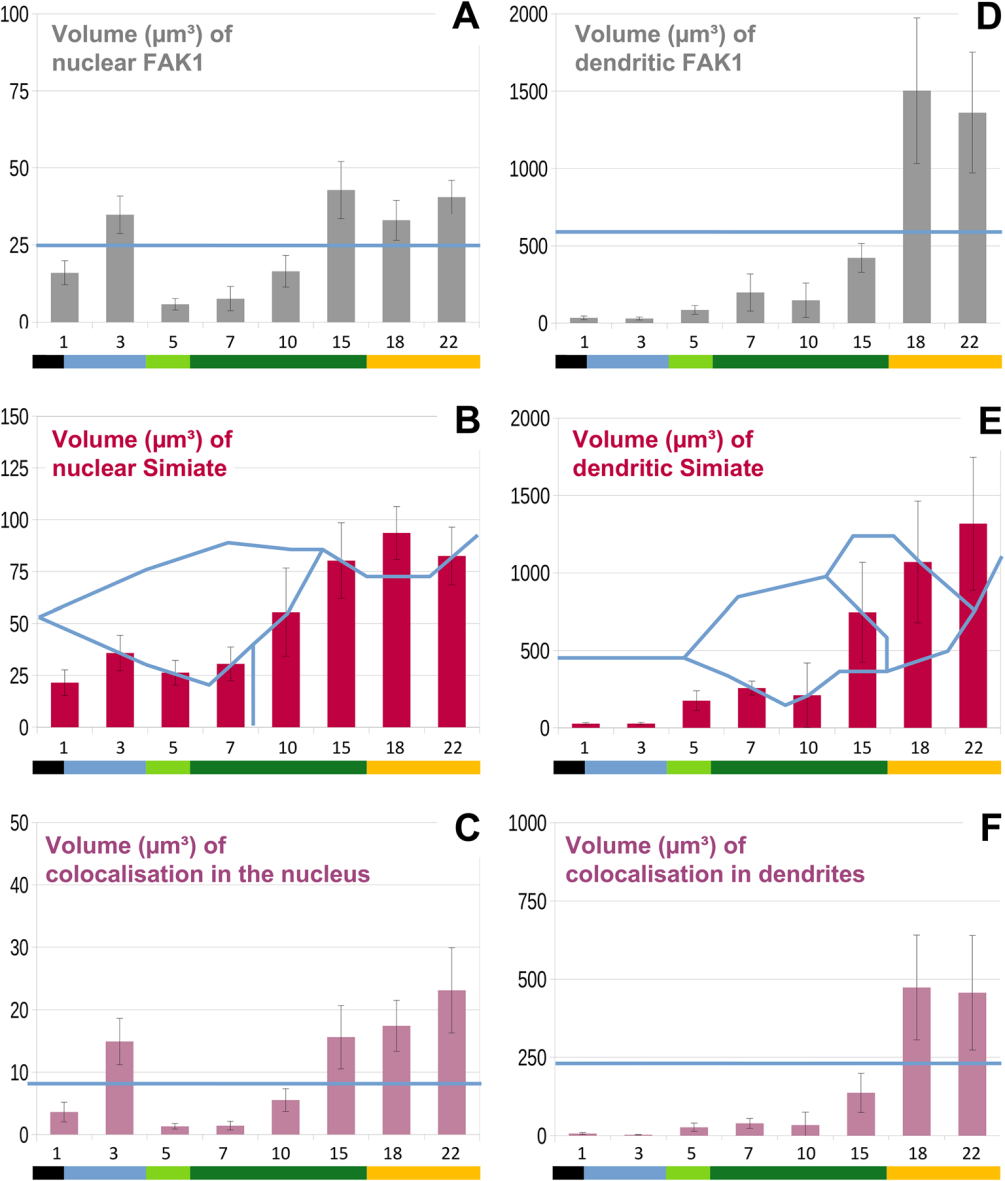
During neuronal development, FAK1 and Simiate display significant variations in their expression levels and their levels of colocalisation in both, nuclei and dendrites. (**A**–**F**) At several developmental stages between div 1 and div 22, the volumes of FAK1 and Simiate as well as the volumes of proteins colocalised were measured in nuclei (**A**–**C**) and dendrites (**D**–**F**) of primary hippocampal neurons by performing 3-dimensional reconstructions. (**A**, **B**) The graphs illustrate the expression of nuclear FAK1 and Simiate. (**C**) Nuclear Simiate and FAK1 show significant changes in their colocalisation during early neuronal development. (**D**, **E**) The expression of FAK1 and Simiate in dendrites varies significantly during development. (**F**) In dendrites, the colocalisation of FAK1 and Simiate rises significantly past div 18. In all graphs, blue lines indicate significance borders. Two mean values are significantly different if they are completely separated by at least one blue line, n equals 8–12 in all experiments. Coloured bars indicate the approximate dendritic stage. Black: protrusions indifferent. Blue: axon growth. Green: onset of dendritogenesis and dendritogenesis. Yellow: slowing of dendritogenesis. Please note that for technical reasons, the total volumes of dendritic proteins were calculated based on densities and known dendrite lengths. For further details, please also see Fig. 4.

Quantifying the volume of FAK1 and Simiate in neurons from div 1 to div 22 by 3-dimensional reconstructions, we found a significant increase in the volume of somatic FAK1 from app. 40 to 100 µm^3^ between div 15 and 18 (Suppl. Table 1 -"Development Text") and variable volumes of somatic Simiate (Suppl. Table 1 -"Development Text"). In the nucleus (Fig. 3A–C and Suppl. Table 1), we observed high levels of FAK1 at div 3 (~ 35 µm^3^) and after div 15 (~ 40 µm^3^), while a significant drop was seen around div 5 to div 7 (Fig. 3A). By contrast, Simiate displayed a significant increase in nuclear protein levels, ranging from about 30 µm^3^ to app. 80 µm^3^ around div 15 (Fig. 3B). Following div 15, both proteins had reached their mature levels in the nucleus. Looking at the volume of colocalised proteins (Fig. 3C), the pattern tightly mirrors the behaviour of FAK1, implying that the interaction of FAK1 and Simiate is associated with FAK1 localisation.

Next, we aimed to analyze the expression of FAK1 and Simiate in dendrites (cp. Fig. 2A/B). Since it is technically not possible to image the entire dendritic tree of maturing neurons and perform detailed 3-dimensional reconstructions at the same time, we calculated the total volume of FAK1 and Simiate (Fig. 3D–F) from reconstructions of proximal dendrites and known total dendrite lengths of cultured primary hippocampal neurons^33–35^. The results revealed that FAK1 levels (Fig. 3D) gradually increase during the first two weeks of development, ranging from about 30 µm^3^ at div 1 to approximately 400 µm^3^ at div 15. Thereafter, a significant rise to about 1500 µm^3^ is seen, which stabilizes at div 22. Simiate levels (Fig. 3E) also display a significant increase, but the rise is more moderate, although final protein levels are similar to FAK1 levels (Suppl. Table 1 -"Development Text"). Indeed, the volumes of dendritic FAK1 and Simiate are comparable throughout development, whereas the volumes of nuclear FAK1 are always significantly lower than those of nuclear Simiate (Suppl. Table 1 -"Development Text") except for div 1 and 3, where no significant difference in the nuclear volumes of both proteins is found (Suppl. Table 1 -"Development Text"). Looking at the volumes of colocalised FAK1 and Simiate in dendrites (Fig. 3F), a significant increase is seen from about 135 µm^3^ at div 15 to about 475 µm^3^ at div 18, whereafter the level remains stable.

**Figure 4:**
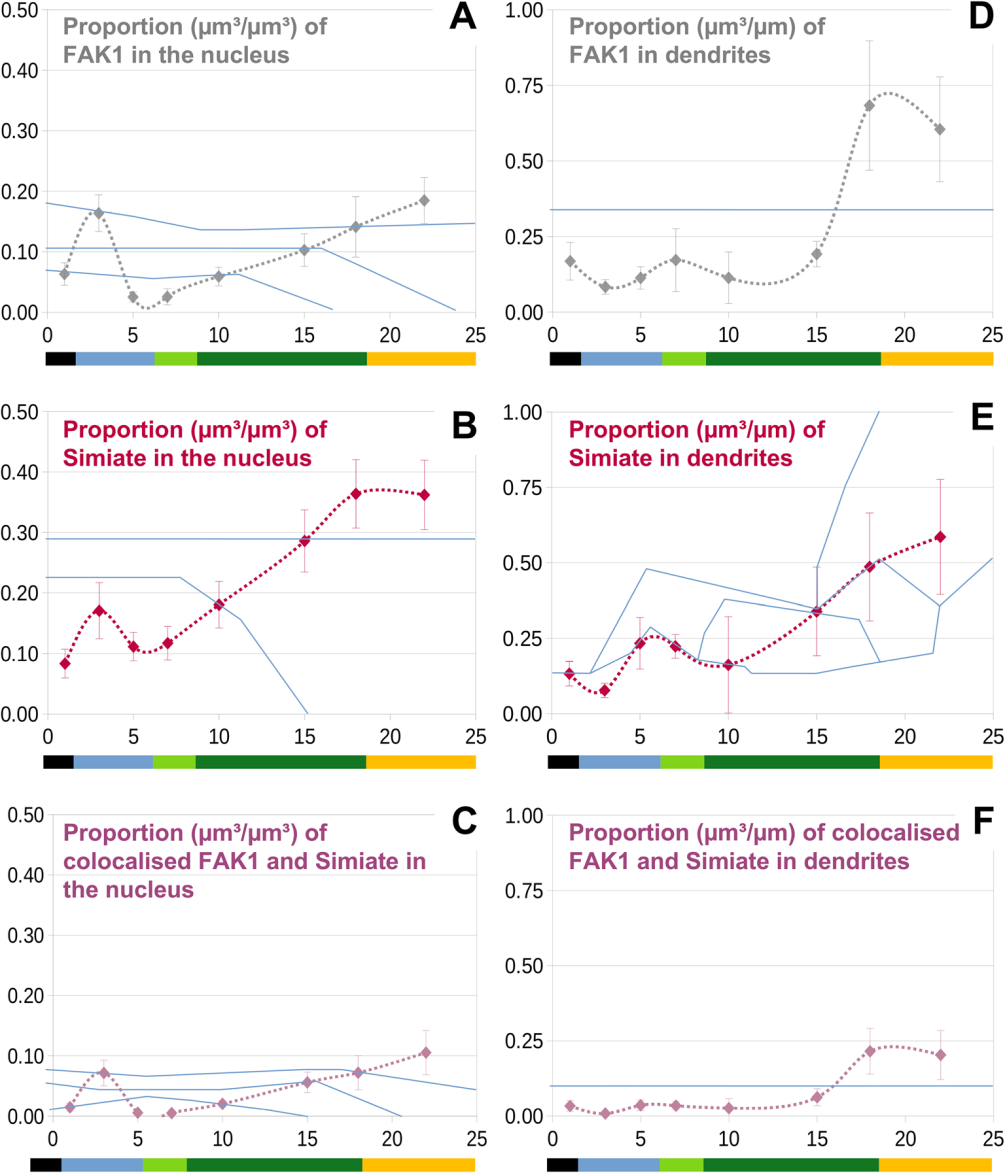
In both, nuclei and dendrites, the proportion of FAK1 and Simiate as well as of their proteins colocalised changes significantly during development. (**A**–**F**) At several developmental stages between div 1 and div 22, primary hippocampal neurons were analysed for the proportions of FAK1, Simiate and their proteins colocalised in nuclei (**A**–**C**; µm^3^/µm^3^) as well as in dendrites (**D**–**F**; µm^3^/µm). The results show significant changes in the proportions of FAK1 and Simiate during development, thus illustrating that levels of FAK1 and Simiate are regulated independently from cellular growth (cp. Fig. 3). For the different units in the measurements of nuclei (per volume: µm^3^) and dendrites (per length: µm) numbers do not compare directly though (please also see Fig. 2A/B). In all graphs, blue lines indicate significance borders. Two mean values are significantly different if they are completely separated by at least one blue line, n equals 8–12 in all experiments. Coloured bars indicate the approximate dendritic stage. Black: protrusions indifferent. Blue: axon growth. Green: onset of dendritogenesis and dendritogenesis. Yellow: slowing of dendritogenesis.

Given that cellular growth may have a considerable impact on the total volume of a protein, we also calculated the proportions of FAK1 and Simiate for nuclei and dendrites (Fig. 4A–C, in µm^3^/µm^3^, and D-F, in µm^3^/µm). Reflecting our previous results on the volume of FAK1 and Simiate (Fig. 3), we found a significant peak in the concentration of nuclear FAK1 (Fig. 4A) at div 3, which coincides with a tendency for elevated levels of nuclear Simiate (Fig. 4B) and increased proportions of colocalisation (Fig. 4C). Following div 10, the concentrations of nuclear FAK1 and Simiate gradually increase until mature levels are reached at about div 22 (Fig. 3A–C).

In dendrites, the proportions of FAK1 (Fig. 4D) and Simiate (Fig. 4E) remain low until div 10, whereafter their concentrations rise until the mature proportion is achieved at about div 22. Again, the rise coincides with increased concentrations of colocalisation after div 18 (Fig. 4C,F). Taken together, the results thus show that neither dendrite growth nor nucleus size can account for the alterations of FAK1 and Simiate levels in nuclear and dendritic compartments. It therefore seems likely that the changes in FAK1 and Simiate are not a consequence of cell growth, but of functional relevance.

### FAK1 and simiate regulate distinct aspects of dendritogenesis

Interestingly, the colocalisation of FAK1 and Simiate in the nucleus is low at div 1, when unspecified neurites are formed (cp.^35^), and at div 5–7, when the initial development of the dendritic tree takes place. Highest levels of colocalisation are seen at div 3, the time of axonal outgrowth (cp.^35^), as well as after div 15, when dendrites mature and synapses develop. The timing observed is in line with the detailed description of neuronal development given by Dotti et al. and is characteristic to neurons in Banker's cultures (reviewed in^36^). Considering the dendrite promoting role of FAK1 and Simiate^13^, the colocalisation of both proteins in the nucleus around div 3 and after div 14 implies that the association serves to halt the dendritic development during axon specification as well as after primary dendrite outgrowth.

Desiring to test this hypothesis, we opted to approach the crux by enhancing the expression levels of our proteins of interest, since earlier studies had shown that FAK1 depletion is associated with apoptosis^37,38^ and that a reduction in Simiate triggers cell death within only hours^12^. Given that dendritic retraction is one of the very first reaction in apoptosis, such a condition would subvert any analysis on dendritogenesis. Thus, we designed a GFP-NLS-Simiate construct to specifically increase Simiate levels in the nucleus, and performed Sholl analyses comparing simultaneous and single expression of FAK1 and Simiate constructs at the time of lowest endogenous colocalisation in the nucleus, div 7, in order to dissect the role of their localisation in dendritogenesis.

#### Recombinant FAK1 and Simiate display distinct effects on the localisation of endogenous Simiate and FAK1

**Figure 5:**
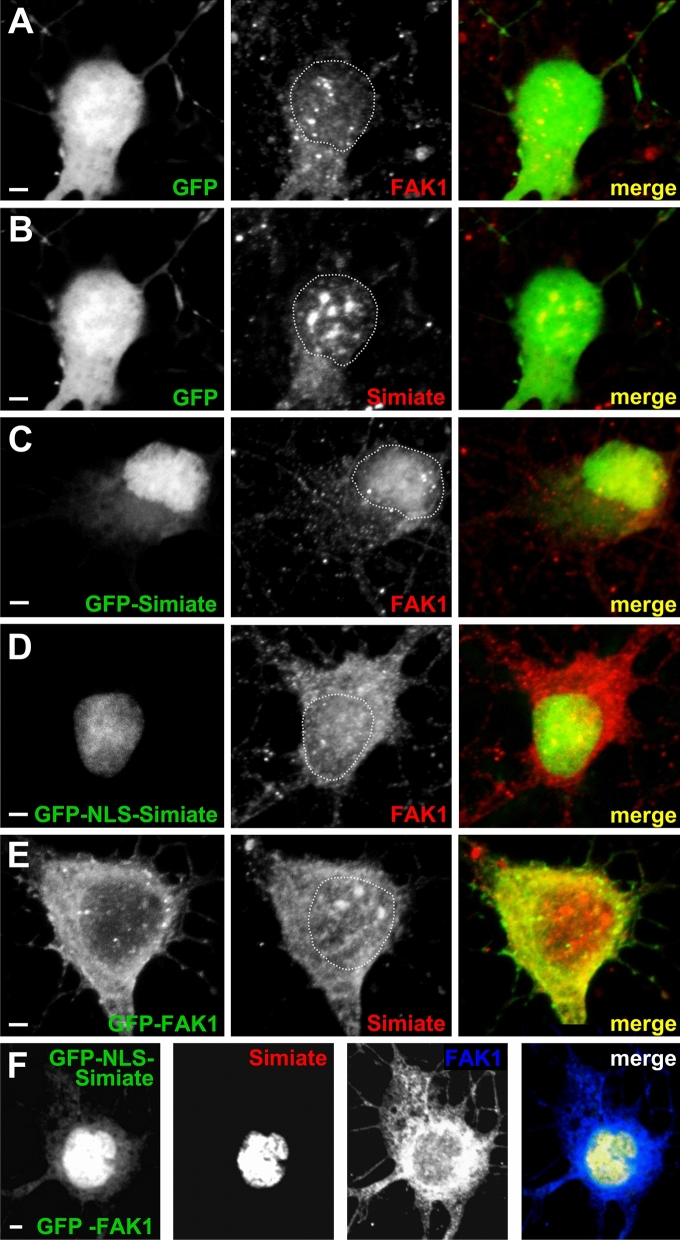
Recombinant FAK1 or Simiate attract endogenous Simiate and FAK1 in a site-specific manner. The pictures show div 8 primary hippocampal neurons after over-night expression of the indicated GFP constructs (green). (**A**, **B**) GFP-expression does not affect the localisation of endogenous FAK1 and Simiate in a detectable manner. As under natural conditions, FAK1 is mainly present in the soma and shows only small amounts in the nucleus, whereas Simiate is seen in the soma and the nucleus, concentrating in nuclear speckles. (**C**) Similar to endogenous Simiate, GFP-Simiate resides in the soma as well as in the nucleus (to varying extents, the picture features a pronounced nuclear localisation). In addition, GFP-Simiate drags endogenous FAK1 into the nucleus, thereby even depleting somatic FAK1. (**D**) Expression of GFP-NLS-Simiate also attracts endogenous FAK1 into the nucleus (cp. **A**). A comparison with C demonstrates that the extent depends on the level of nuclear Simiate and not on its presence at other sites (cp. **C**). (**E**) Following the expression of GFP-FAK1, endogenous Simiate is seen in the nucleus, where it is present in nuclear speckles, reflecting control conditions (cp. **B**). (**F**) Following a co-expression of GFP-FAK1 and GFP-NLS-Simiate, a colocalisation of both proteins ins seen in the nucleus. The recombinant proteins were distinguished by counterstaining FAK1 and Simiate with antibodies specific to these proteins. Scale bars: 2 µm. The images are contrast enhanced to demonstrate the colocalisation of FAK1 and Simiate (yellow). For further details, please also see Figs. 6 and 7.

**Figure 6:**
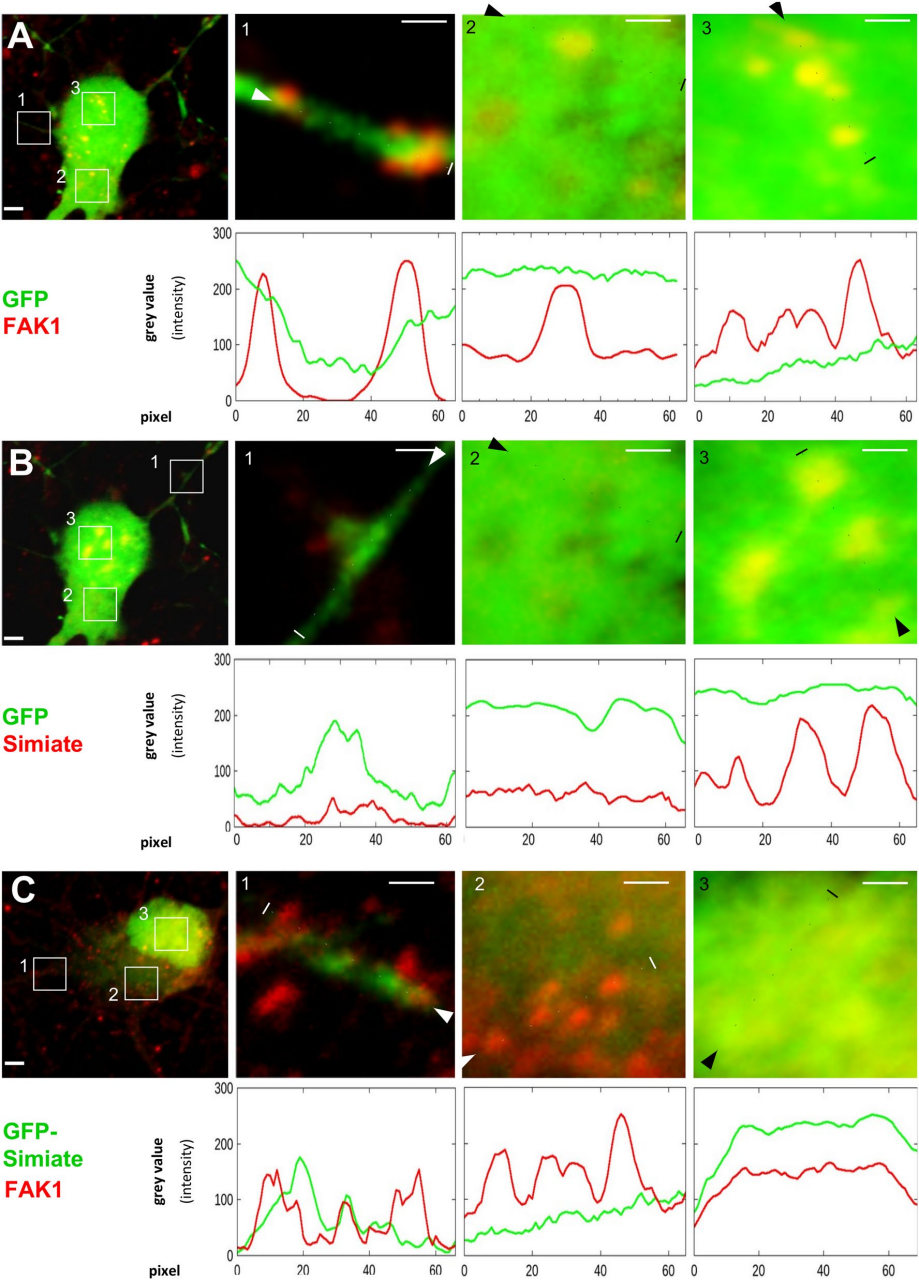
Alterations in the localisation of endogenous FAK1 and Simiate following expression of recombinant constructs (part 1). The pictures show representative micrographs of div 8 primary hippocampal neurons after over-night expression of GFP constructs (cp. Fig. 5). For each condition, a neuron and magnifications of parts from a dendrite, from the soma and from the nucleus are shown. The respective areas are indicated by white boxes in the overview-picture on the left. Below each magnification, graphs illustrate the signal intensities as exemplary quantified with ImageJ software for each protein of interest. The results are derived from 2 to 4 experiments. Dotted lines indicate the line of measurement. Scale bars: 1 µm in the magnifications and 2 µm in the overview picture. All images are contrast enhanced to demonstrate the colocalisation of FAK1 and Simiate (yellow). (**A**,**B**) Expressed GFP shows no colocalisation with FAK1 or Simiate in dendrites, somata or nuclei. (**C**) GFP-Simiate attracts some endogenous FAK1 in dendrites, but not in the soma. A strong attraction is seen in the nucleus. Please also see Fig. 7 for further details on the other contructs.

**Figure 7:**
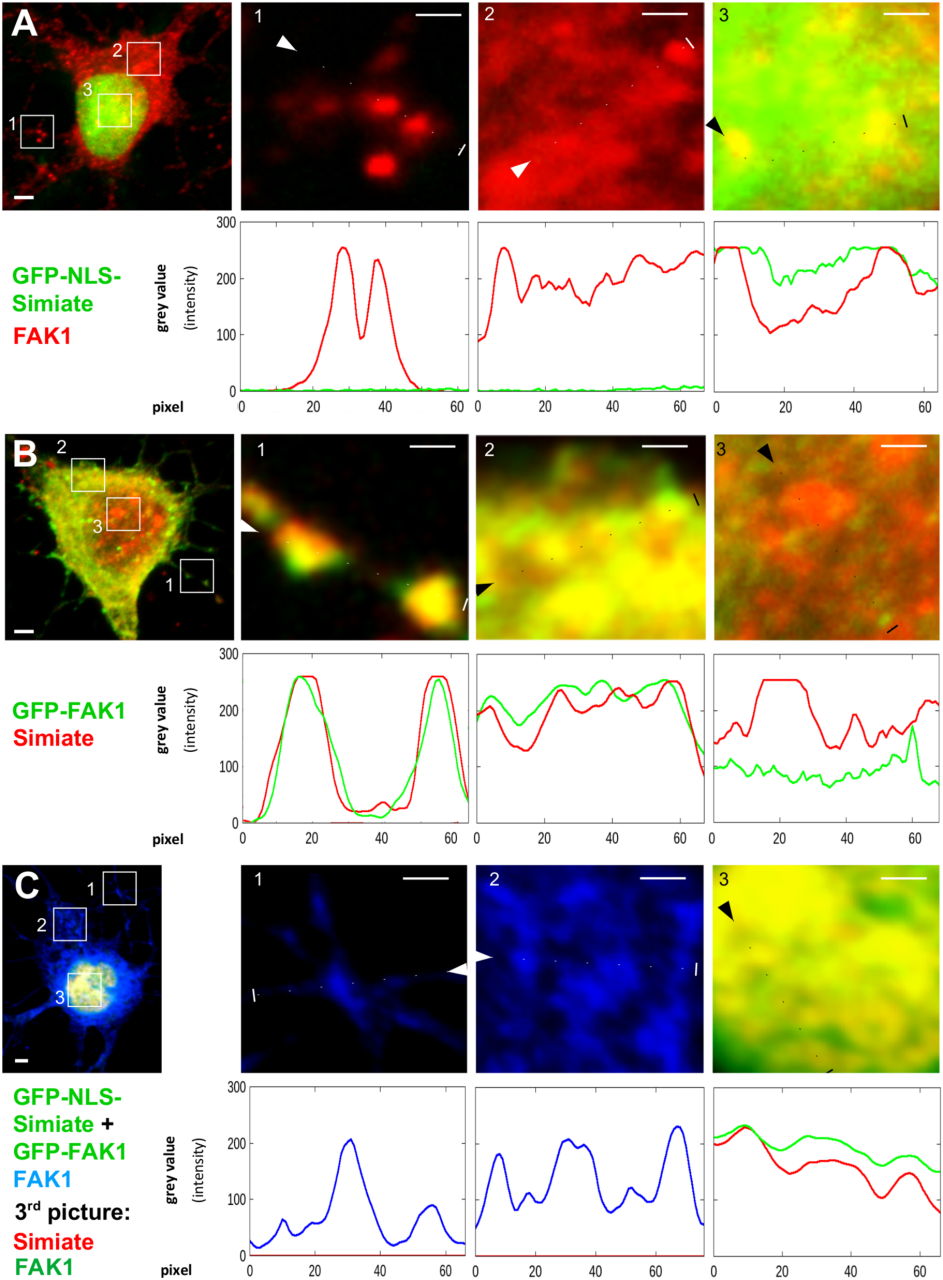
Alterations in the localisation of endogenous FAK1 and Simiate following expression of recombinant constructs (part 2). The pictures show representative micrographs of div 8 primary hippocampal neurons after over-night expression of GFP constructs (cp. Figs. 5 and 6). For each condition, a neuron and magnifications of parts from a dendrite, from the soma and from the nucleus are shown. The respective areas are indicated by white boxes in the overview-picture on the left. Below each maginification, graphs illustrate the signal intensities as exemplary quantified with ImageJ software for the proteins of interest. The results are derived from 2 to 4 experiments. Dotted lines indicate the line of measurement. Scale bars: 1 µm in the magnifications and 2 µm in the overview picture. All images are contrast enhanced to demonstrate the colocalisation of FAK1 and Simiate (yellow). (**A**) GFP-NLS-Simiate neither shows any signal in dendrites nor in the soma, whereas in the nucleus, a strong expression is seen. Here, the construct shows some colocalisation with endogenous FAK1. In order to avoid areas of saturated GFP-NLS-Simiate signal, the measurement for the nucleus was performed in a curve as indicated by the dotted line. (**B**) GFP-FAK1 colocalises with endogenous Simiate in dendrites and somata, but not in the nucleus. (**C**) The recombinant proteins were distinguished by counterstaining FAK1 and Simiate with antibodies specific to these proteins. While no GFP-NLS-Simiate is seen in somata or dendrites, a high expression is found in the nucleus. Here, a significant colocalisation with FAK1 is observed. For reasons of clarity, the last magnification displays FAK1 in green (false colour). Please note that the endogenous Simiate signal is too weak to be detected during expression of GFP-NLS-Simiate.

First, we examined the localisation of our constructs in primary hippocampal neurons (Figs. 5, 6 and 7). Though GFP is found throughout the cells, the protein enriches in nuclei, probably due to its bipartite nuclear localisation signal (Fig. 5A,B). While GFP-Simiate is also seen in somata and nuclei (Fig. 5C), GFP-NLS-Simiate is confined to the nucleus (Fig. 5D). By contrast, GFP-FAK1 is mainly present in the soma, although some GFP-FAK1 is found in the nucleus as well (Fig. 5E). The localisations of FAK1 and Simiate are in line with our fractionation experiments (Fig. 1B, E–I), which demonstrated the proteins to be present in cytosolic as well as nuclear fractions. Co-expressing GFP-FAK1 and GFP-NLS-Simiate (Fig. 5F), both proteins show their expected localisation, with enhanced levels of FAK1 being present in the soma and the nucleus.

Given the association and colocalisation of Simiate and FAK1 (Figs. 1, 2, 3, 4, 5), we also assessed a possible influence of our constructs on the localisation of endogenous FAK1 or Simiate (Figs. 6 and 7). GFP served for control (Fig. 6A,B). Interestingly, the experiments revealed that the constructs affect endogenous FAK1 and Simiate in distinct ways: while GFP-Simiate attracts some endogenous FAK1 in dendrites and nuclei, but not in somata (Fig. 6C), GFP-FAK1 significantly enriches endogenous Simiate in dendrites and somata (Fig. 7B versus Fig. 6B, magnifications), but not in nuclei (Fig. 7B, magnifications). GFP-Simiate (Fig. 6C) and GFP-NLS-Simiate (Fig. 7A) both attract endogenous FAK1 in the nucleus (Figs. 6C and 7A versus 6A,B), while somatic GFP-Simiate does not significantly enrich endogenous FAK1 (magnifications in Fig. 6C versus Fig. 6A). Although high levels of nuclear Simiate may cause a depletion of endogenous FAK1 in the soma (Fig. 5C (also see 5D) versus 5A), GFP-FAK1 is not able to deplete endogenous Simiate in the nucleus (Fig. 5E versus 5B). Looking at the co-expression of GFP-FAK1 and GFP-NLS-Simiate (Figs. 5F and 7C), it turns out that both proteins colocalise in the nucleus. These findings strongly support an association of FAK1 and Simiate in vivo, and further suggest that the interaction is strictly regulated (cp. Fig. 1).

#### Simiate and FAK1 alter dendritic arborisation in different ways, but are able to counteract each other when affecting proximal dendrites

**Figure 8:**
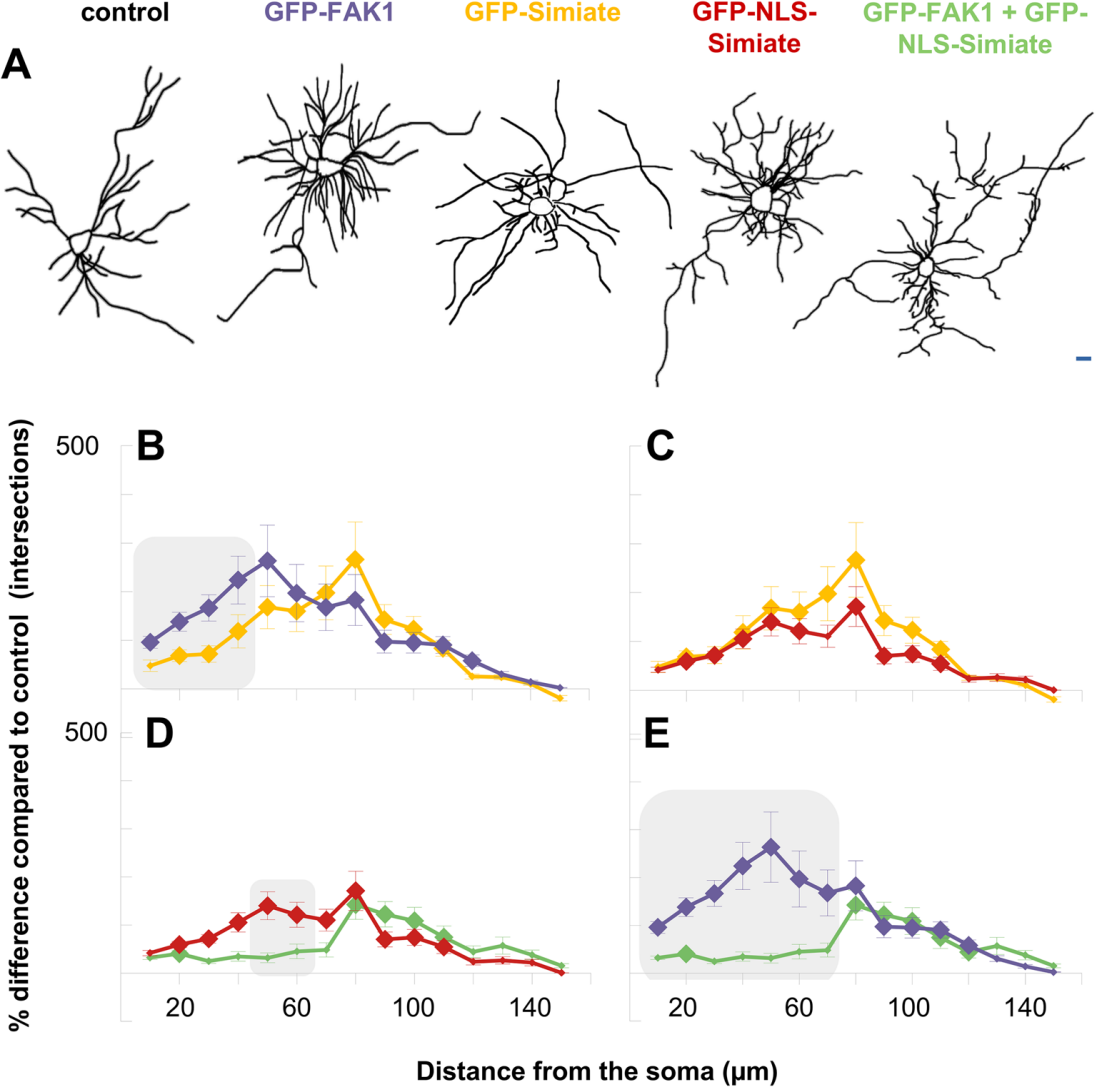
FAK1 and Simiate function together in dendritogenesis. (**A**) Dendritic trees experience different degrees of complexity after over-night expression of recombinant FAK1 and Simiate. Neurons expressing the indicated constructs were processed in Fiji software for Sholl analyses based on MAP2 staining and GFP signal. (**B**–**E**) Results from Sholl analyses are shown as percentage difference to GFP expression (control). Significant changes to control conditions are indicated by big rhombs, whereas significant differences between constructs are displayed by grey boxes. Small rhombs indicate no significant difference from control, as for instance seen with Simiate constructs (yellow and red lines) beyond 120 µm or in the co-expression experiments (green lines) around 50 µm. Scale bar: 10 µm. GFP: n = 20; FAK1: n = 21; Simiate: n = 21; NLS-Simiate: n = 21; FAK1 + NLS-Simiate: n = 18. For more statistical details, please see Suppl. Table 1.

Next, we examined the effects of our FAK1- as well as Simiate constructs on the morphology of primary hippocampal neurons (Fig. 8). GFP served for control. In order to visualise the dendritic tree, we employed co-stainings with MAP2, a marker protein specifically labelling dendrites, and the respective GFP signals (Fig. 8A). The analyses revealed that GFP-FAK1 as well as GFP-Simiate enhance dendrite formation in primary hippocampal neurons (Fig. 8A), thereby reaffirming previous results^13,26^. Interestingly though, GFP-NLS-Simiate is also able to enhance dendrite formation, whereas a co-expression of GFP-NLS-Simiate and GFP-FAK1 causes only a mild increase in arborisation (Fig. 8A).

In order to investigate our observations in more detail, we performed Sholl analyses (Fig. 8B–E and Suppl. Table 1). Comparing the effects of GFP-FAK1 and GFP-Simiate, GFP-FAK1 enhances the dendritic complexity significantly more than GFP-Simiate, but only between 10 and 40 µm distance from the soma. Beyond 50 µm, GFP-FAK1 and GFP-Simiate do not show any significant differences (Fig. 8B), although both constructs further increase the complexity of distal dendrites.

Looking at GFP-Simiate and GFP-NLS-Simiate, no divergences in their effects on dendritic complexity are found (Fig. 8C), thus showing that nuclear Simiate is sufficient to enhance neuronal arborisation. Remarkably though, a co-expression of GFP-NLS-Simiate and GFP-FAK1 does not further increase dendritic complexity, but instead results in a restoration of control complexity levels between 30 and 60 µm (Fig. 8D,E), whereas distal to 60 µm the enhanced dendritic arborisation characteristic to both constructs is seen. This reconstruction of normal complexity implies that FAK1 and Simiate are able to counteract each other in order to control the complexity of dendrites.

#### Dendritic length and branching are altered differentially by FAK1 and Simiate

**Figure 9:**
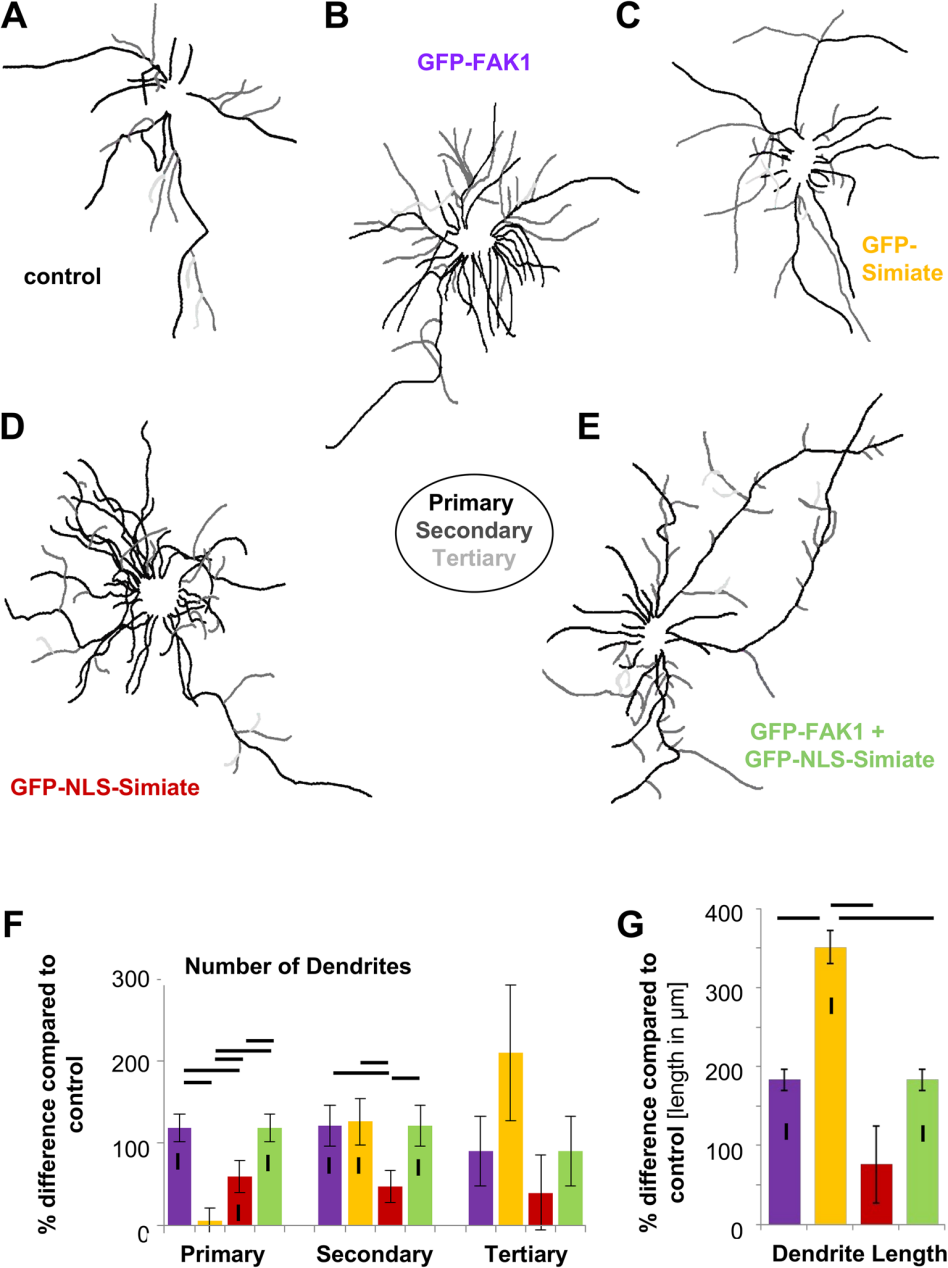
FAK1 and Simiate have different effects on primary, secondary and tertiary dendrites. Following over-night expression of recombinant proteins, div 8 primary hippocampal neurons were imaged and processed for arborisation analyses in NeuronJ, a plug-in for Fiji software. (**A**–**E**) The pictures illustrate primary (black), secondary (grey) and tertiary (light grey) dendrites. (**F**,**G**) Number (**F**) and average length (**G**; measured in µm) of dendrites are shown as percentage difference from GFP. While vertical bars indicate a significant deviation from control conditions (zero level), horizontal bars display significant differences between constructs. For all constructs, n equals 10. Please refer to Suppl. Table 1 for further statistical details.

Since Sholl analyses do not directly distinguish between alterations in dendrite length and branching, we surveyed primary, secondary and tertiary dendrites separately (Fig. 9 and Suppl. Figure for Fig. 9). The quantifications revealed that all constructs act on primary and/or secondary dendrite numbers, whereas no significant effects on tertiary dendrites are seen (Fig. 9A–F and Suppl. Table 1). While GFP-FAK1 equally elevates the number of primary and secondary dendrites (Fig. 9B,F), GFP-Simiate increases the number of secondary dendrites only (Fig. 9C,F). By contrast, GFP-NLS-Simiate significantly intensifies the number of primary dendrites (Fig. 9D,F).

Comparing the action of FAK1 and Simiate (Fig. 9F), it turns out that GFP-FAK1 enhances the number of primary dendrites significantly more than GFP-Simiate and GFP-NLS-Simiate, but about as much as under co-expression with GFP-NLS-Simiate. By contrast, the number of secondary dendrites is equally affected by GFP-FAK1, GFP-Simiate and a co-expression of both proteins, only GFP-NLS-Simiate shows a significantly smaller increase in the number of secondary dendrites.

Measurements of dendrite lengths (Fig. 9G and Suppl. Table 1) revealed that all constructs increase dendrite length except for GFP-NLS-Simiate, which does not show any differences from control conditions in this regard. Remarkably though, GFP-Simiate displays the most pronounced increase in dendrite length, whereas the effect of GFP-FAK1 is significantly smaller and almost identical to the one obtained by co-expression of GFP-FAK1 and GFP-NLS-FAK1. Dendrite length is hence neither significantly influenced by nuclear Simiate, nor controlled by a balanced expression of FAK1 and Simiate in the nucleus, but instead greatly increased by somatic Simiate.

#### The effects of FAK1 and Simiate depend on dendrite order and distance to the soma

**Figure 10:**
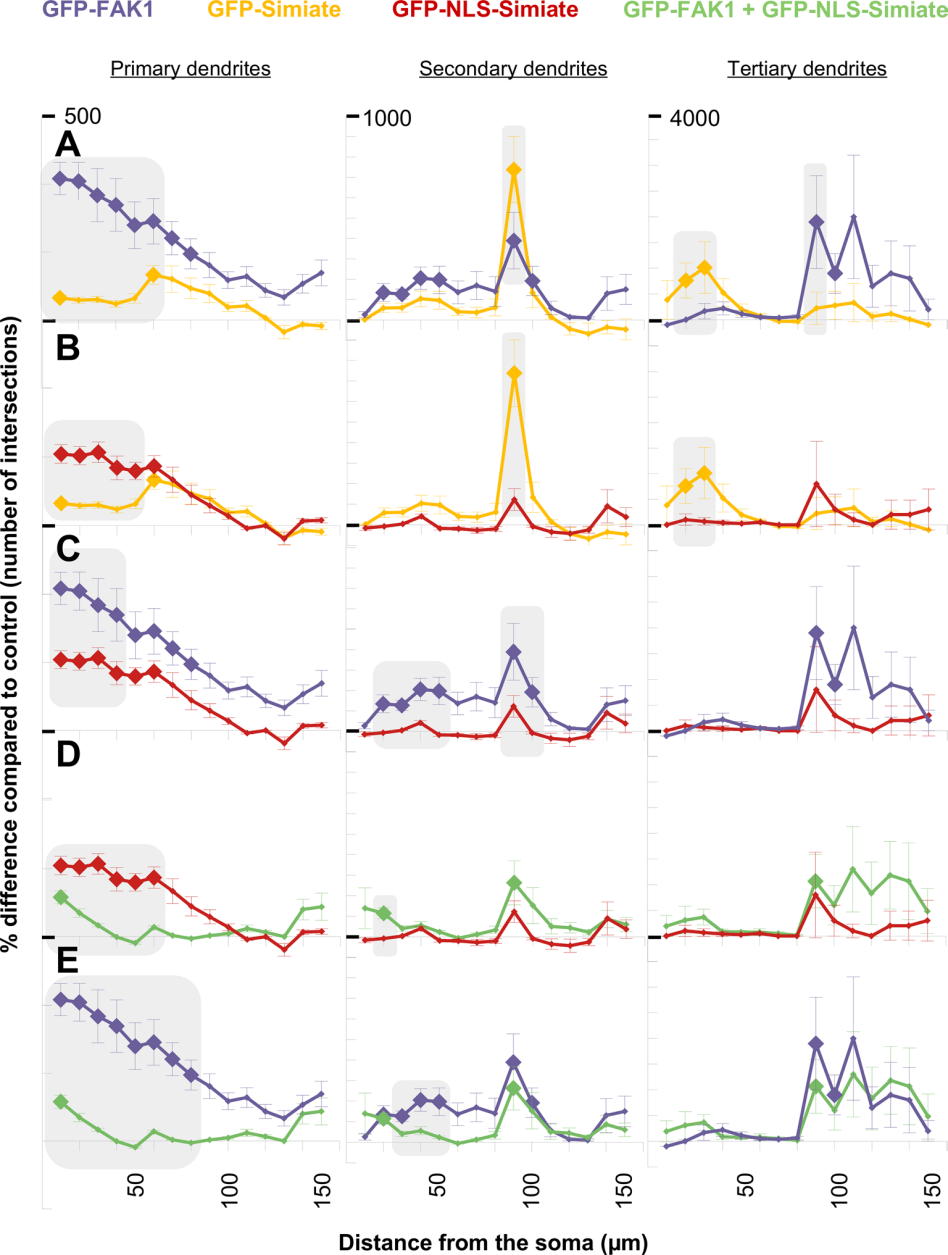
Sholl analyses of primary, secondary and tertiary dendrites reveal Simiate and FAK1 to influence neuronal arborisation in distinct ways. In all graphs, big rhombs indicate changes significantly different from control (GFP expressing neurons), while grey boxes highlight significant differences between constructs. Please note the different scaling between primary (500% maximum deviation), secondary (1000% maximum deviation) and tertiary dendrites (4000% maximum deviation). (**A**–**E**) Constructs are compared according to dendrite order and distance to the soma. For all constructs, n equals 12–14. Further statistical details are shown in Suppl. Table 1.

It is noteworthy that no amelioration of dendritic arborisation is seen in any of the analyses shown in Fig. 9E–G. Given that the previously observed renormalisation effect was limited to proximal dendrites, the results thus suggest that the distance to the soma is crucial to the outcome. In order to verify this finding, we performed Sholl analyses on primary, secondary and tertiary dendrites separately (Fig. 10 and Suppl. Table 1). The results show that the effects of FAK1 and Simiate indeed depend on dendrite order *and* distance to the soma:

GFP-FAK1 enhances primary and secondary dendrite formation prior to 50 µm of distance, but GFP-FAK1 also significantly augments secondary dendrites at 90–100 µm, as well as tertiary dendrites beyond 90 µm of distance (Fig. 10A). No effect is seen though on primary dendrites beyond 90 µm, on secondary dendrites beyond 110 µm and on tertiary prior to 90 µm of distance.

Looking at GFP-Simiate, the protein shows only mild elevations in proximal primary dendrite complexity, however, a sharp and significant increase in secondary dendrite complexity is seen at 90 µm of distance. Moreover, significantly elevated levels of tertiary dendrite complexity are found at 30 µm of distance (Fig. 10A,B), where primary dendrites typically dominate.

Comparing GFP-Simiate and GFP-NLS-Simiate, it turns out that the number of proximal primary dendrites achieved by GFP-NLS-Simiate is significantly higher than the number achieved by GFP-Simiate (Fig. 10B), a finding that further supports our previous results on dendrite numbers (Fig. 9F). Indeed, the GFP-Simiate specific increase of tertiary dendrites at 30 µm of distance explains why overall Sholl analyses (Fig. 8) detected no difference for GFP- and GFP-NLS-Simiate despite the higher impact of NLS-Simiate on proximal primary dendrites.

GFP-NLS-Simiate and GFP-FAK1 also display significant differences in their effects (Fig. 10C), with GFP-FAK1 showing more profound increases in primary dendrite complexity than GFP-NLS-Simiate. In addition, GFP-FAK1 significantly enhances secondary dendrite development (Fig. 10A,C,E), whereas GFP-NLS-Simiate hardly affects secondary dendrites at all (Fig. 10B–D).

When looking at the co-expression of GFP-FAK1 and GFP-NLS-Simiate, it turns out that the normalisation effect noted before (Fig. 8E) is limited to proximal primary dendrites (Fig. 10D,E), thus suggesting that changes in more distal areas may have covered the effect in summarized measurements (cp. Fig. 9). Indeed, the specificity of the normalisation effect also indicates that it is the function of nuclear Simiate, which is blocked by FAK1 expression: If a loss of FAK1 in the soma and the dendrites (cp. Fig. 5) would be underlying the renormalisation, the entire dendritic tree and in particular distal secondary and tertiary dendrites should show a restoration of regular complexity, but this is not the case (cp. Fig. 10C,E), instead the formation of primary dendrites, which is characteristic to nuclear Simiate, is brought back to normal. Taken together, these results demonstrate that FAK1 and Simiate differentially affect primary, secondary and tertiary dendrites: while enhanced expression of FAK1 increases dendrite formation and branching in accordance with the structure of dendritic trees, Simiate causes early tertiary dendrite formation or enhanced primary dendrite production if confined to the nucleus. Further, the effect of nuclear Simiate on primary dendrites is specifically renormalized by a co-expression of FAK1, while its effect on tertiary dendrite formation remains unchanged, thus showing that the interaction of FAK1 and Simiate may function to specifically regulate primary dendrite formation in developing hippocampal neurons.

### FAK1 and Simiate regulate transcriptional processes

**Figure 11:**
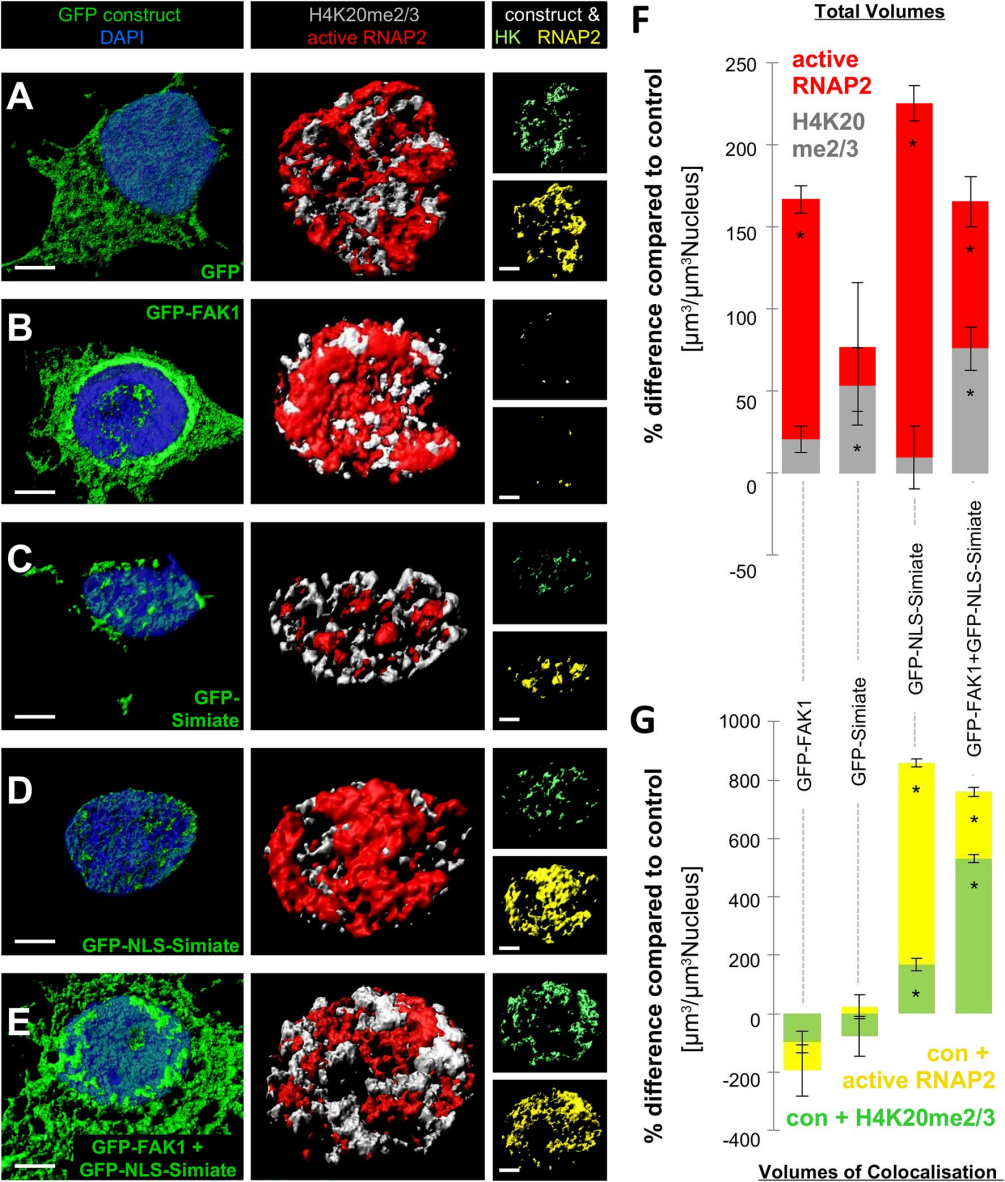
FAK1 and Simiate alter transcription activity. Using Imaris software, 3-dimensional reconstructions of recombinant proteins, active RNAP2, H4K20me2/3 (HK), a marker for inactive chromatin, and proteins colocalising were performed. (**A**–**E**) Three-dimensional reconstructions of H4K20me2/3 and RNAP2 illustrate alterations in the volumes of both marker proteins following enhanced expression of FAK1 and Simiate. Left column: expression of constructs. Middle column: expression of marker proteins. Right column: intersections of the respective GFP construct and marker proteins. (**F**, **G**) Quantifications of corresponding protein volumes are shown as deviation from GFP conditions. The graphs illustrate the total volume of active RNAP2 and H4K20me2/3 (**F**) and the amount of colocalisation with GFP constructs (**G**). Since recombinant protein expression in itself may influence gene expression activity, GFP expression serves as control. Statistical details are summarised in Suppl. Table 1. Scale bars: 3 µm. Con: construct (GFP-FAK1, GFP-Simiate and GFP-NLS-Simiate). GFP: n = 8; FAK1: n = 8; Simiate:n = 8; NLS-Simiate: n = 11; FAK1 + NLS-Simiate: n = 8. Colour coding: blue—DAPI, green—GFP or GFP construct, grey—H4K20me2/3, red—active RNAP2; light green—H4K20me2/3 colocalised with GFP constructs, yellow—active RNAP2 colocalised with GFP constructs.

**Figure 12:**
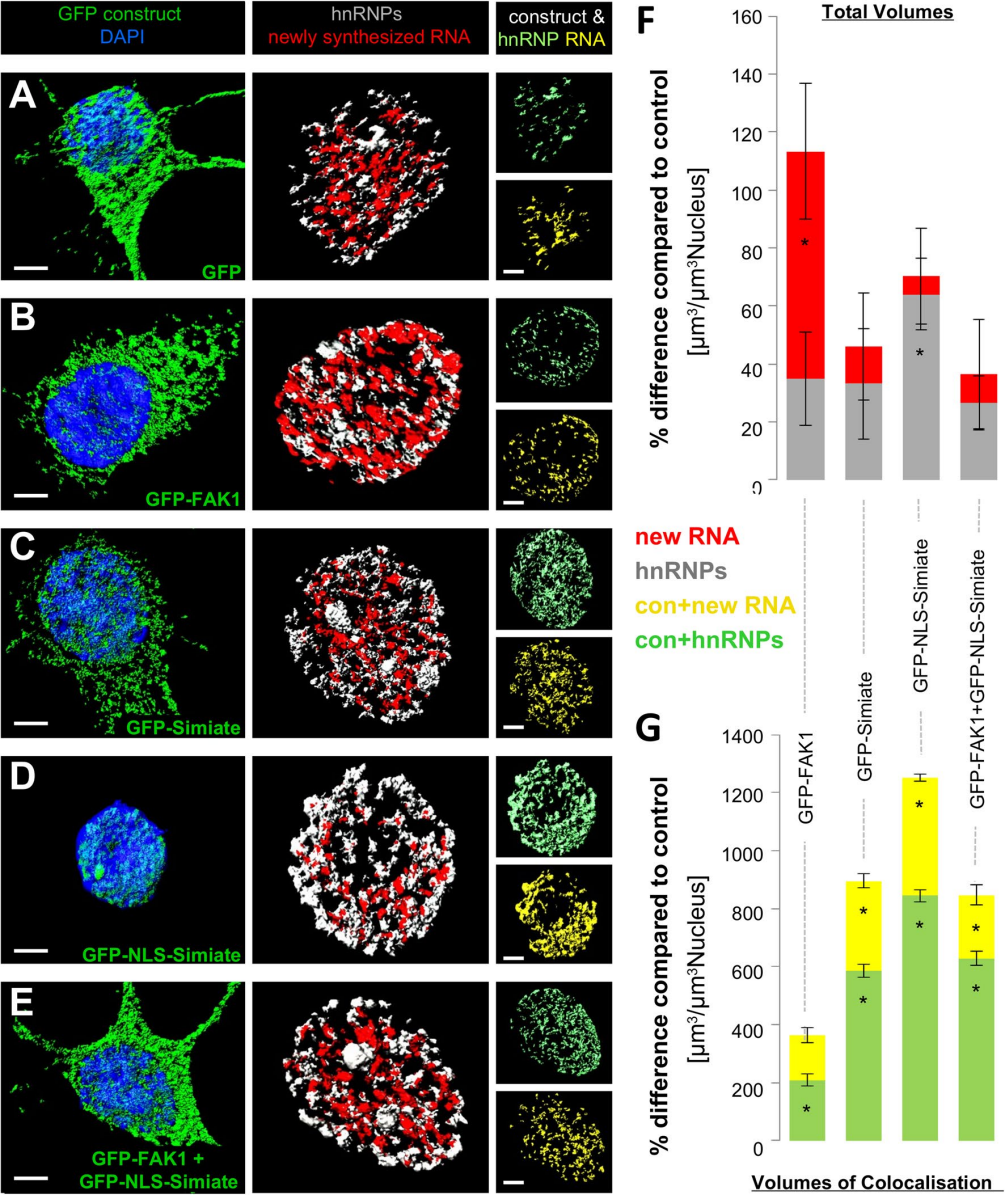
Simiate but not FAK1 affects RNA processing. Recombinant proteins, newly synthesised RNA and hnRNPs, a marker for RNA processing, as well as colocalised proteins were reconstructed 3-dimensionally and quantified in Imaris software. (**A**–**E**) The pictures show reconstructions of hnRNPs and newly synthesised RNA after enhanced expression of FAK1 and Simiate constructs. Left column: expression of constructs. Middle column: expression of marker proteins. Right column: intersections of the respective GFP construct and marker proteins. (**F**, **G**) Quantifications of corresponding volumes (**F**) and volumes of colocalisation (**G**) are displayed as deviation from GFP conditions. Again, GFP serves to control for unspecific effects on transcription and splicing activity caused by recombinant expression. GFP: n = 8; FAK1: n = 7; Simiate: n = 8; NLS-Simiate: n = 8; FAK1 + NLS-Simiate: n = 8. Further statistical details are summarised in Suppl. Table 1. Scale bars: 3 µm. Con: construct (GFP-FAK1, GFP-Simiate and GFP-NLS-Simiate). Colour coding: blue—DAPI, green—GFP or GFP construct, grey—hnRNPs, red -newly synthesized RNA; light green—hnRNPs colocalised with GFP constructs, yellow—newly synthesized RNA colocalised with GFP constructs.

How do FAK1 and Simiate bring about their effects? Considering the colocalisation of FAK1 and Simiate in nuclear speckles (Fig. 2^12,13^), compartments serving as organisation centres for the transcription and splicing machinery (reviewed in^30^), and the role of FAK1 in chromatin remodelling and transcription regulation (reviewed in^17,19^), we set out to study the effects of our recombinant proteins on gene activity (Figs. 11 and 12, Suppl. Table 1). Therefore, we labelled actively transcribing RNA polymerase (active RNAP2) and H4K20me2/3, a marker for inactive Chromatin, after expression of our proteins of interest in primary hippocampal neurons, and reconstructed the proteins 3-dimensionally (Fig. 11A–E). In order to control for unspecific effects, such as increased transcription due to recombinant protein expression, GFP was utilized as reference (Fig. 11A, in Fig. 11F and G represented by baselines).

The experiments revealed significant increases in the volume of active RNAP2 (Fig. 11F, shown in red) following expression of all our constructs, except for GFP-Simiate, which has no such an effect, but rather increases the volume of inactive Chromatin (Fig. 11B–E, middle column, and Fig. 11F, shown in grey). Strikingly, GFP-NLS-Simiate caused the most profound rise in the volume of active RNAP2 (Fig. 11D,F, shown in red).

Looking at the co-expression of GFP-NLS-Simiate and GFP-FAK1, an intermediate effect is seen; both, inactive Chromatin as well as active RNAP2 display an increase in their volumes (Fig. 11E, middle column and Fig. 11F). Interestingly though, compared to GFP-FAK1 and GFP-NLS-Simiate, the volume of active RNAP2 is reduced, while the volume of Heterochromatin is increased (Fig. 11F), thus suggesting that the interaction of nuclear Simiate and FAK1 serves to regulate active transcription. Analysing the colocalisation (Fig. 11B–E, right column, and Fig. 11G), only GFP-NLS-Simiate displays a clear colocalisation with active RNAP2 (Fig. 11G, shown in yellow) and some colocalisation with H4K20me2/3 (Fig. 11G, shown in light green), implying that the effects of GFP-FAK1 and GFP-Simiate are in contrast to those of GFP-NLS-Simiate indirect.

In order to further investigate the function of nuclear FAK1 and Simiate, we next analysed the presence of newly synthesised RNA and hnRNPs, a marker for RNA processing, following expression of our constructs (Fig. 12A–E). In line with the exaggerated transcription observed previously upon GFP-FAK1 expression (Fig. 11B,F, shown in red), the protein now shows a significant increase in the presence of newly synthesized RNA (Fig. 12B, middle column, and Fig. 12F, shown in red). Also GFP-Simiate demonstrates similar results in both experiments, displaying no indication for significantly enhanced transcription activity (Figs. 11C and 12C, middle column, and Figs. 11F and 12F, shown in red). Strikingly though, while the results obtained for GFP-FAK1 and GFP-Simiate reflect the findings received with active RNAP2 and H4K20me2/3, GFP-NLS-Simiate does not enhance the volume of newly synthesised RNA despite its exaggeration of transcription (Figs. 11 and 12F), but instead significantly increases the volume of hnRNPs, the marker for RNA processing (Fig. 12D, middle column, and Fig. 12F). These findings thus suggest that the RNA synthesised upon GFP-NLS-Simiate expression is quickly processed and exported. Indeed, in contrast to FAK1, nuclear GFP-Simiate and GFP-NLS-Simiate show a profound colocalisation with both, newly synthesised RNA and hnRNPs (Fig. 12C,D, right column, and Fig. 12G).

### External signals can cause a functional cooperation of FAK1 and simiate in dendrites

**Figure 13:**
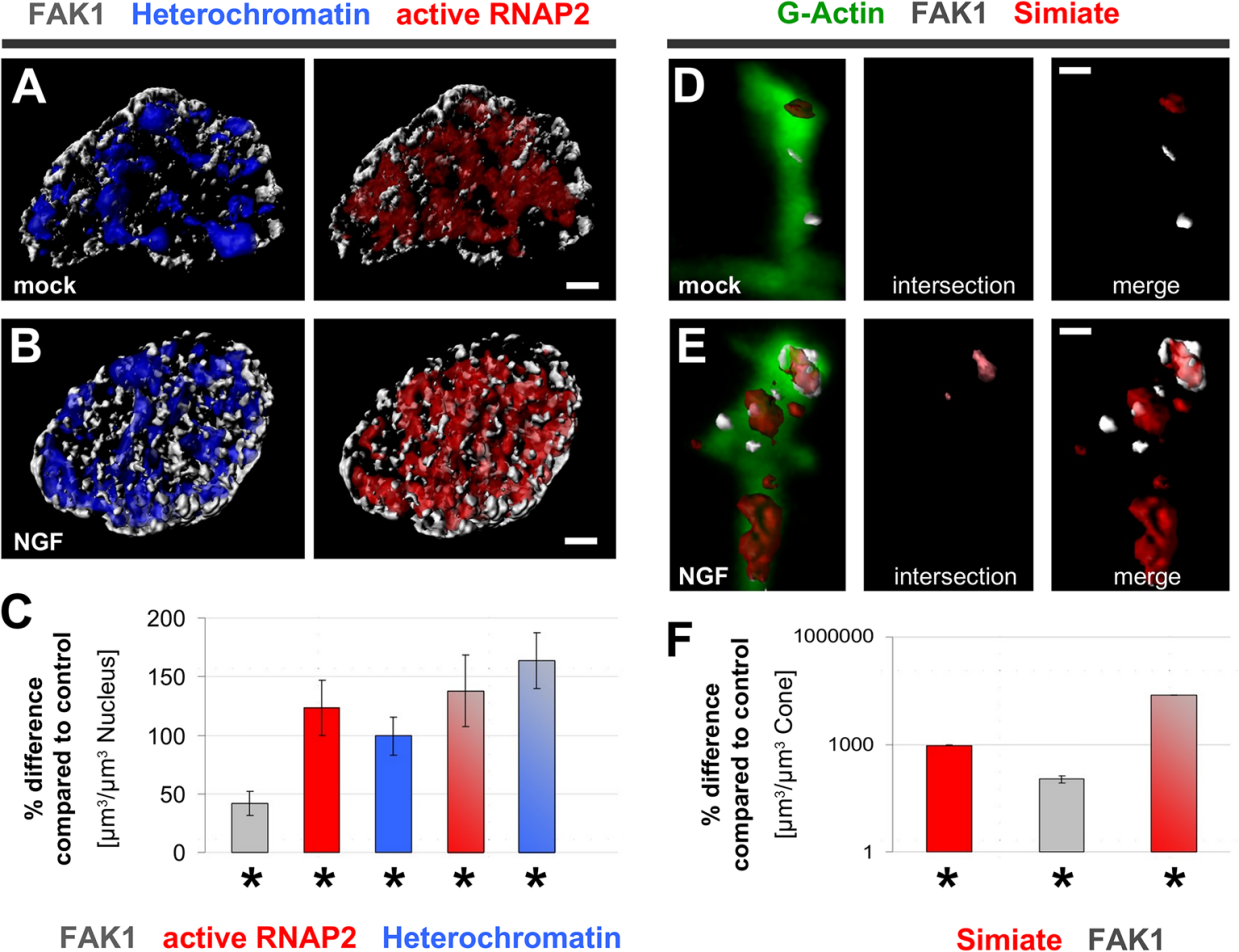
FAK1 and Simiate respond to neurotrophin stimulation. Following over-night treatment with NGF, div 3 primary hippocampal neurons were imaged confocally and analysed in Imaris software. Non-significant effects on FAK1, Simiate, hnRNPs and RNA as well as their colocalisations are shown in Suppl. Table 1. **A**–**C**) NGF application influences FAK1, active RNAP2 and Heterochromatin in the nucleus of hippocampal neurons. (**A**, **B**) Three-dimensional reconstructions of FAK1, Heterochromatin and active RNAP2 in mock treated (**A**) and NGF treated (**B**) cells illustrate increased amounts of FAK1, RNAP2 and Heterochromatin as well as enhanced colocalisations in the nucleus following NGF application. Scale bars: 2 µm. (**C**) A quantification of FAK1 (grey), active RNAP2 (red) and Heterochromatin (blue) volumes as well as of proteins colocalising (grey-red and grey-blue) demonstrates significant increases in all protein volumes. (**D**, **E**) Three-dimensional reconstructions illustrate FAK1 and Simiate in dendritic growth cones following mock (**D**) or NGF (**E**) treatment (middle panel: intersection of colocalised FAK1 and Simiate, light red; right panel: FAK1 and Simiate). G-Actin labelling with DNaseAx488 served to outline the cone structure. Scale bars: 1 µm. (**F**) The volumes of FAK1 (grey), Simiate (red) as well as of colocalised FAK1 and Simiate (red-grey) are shown for dendritic growth cones. For all analyses, n equals 8. More details on the statistics can be found in Suppl. Table 1.

Neurotrophins are important regulators of dendritic development and synaptic plasticity. Given that NGF treatment was previously shown to enhance neurite outgrowth in a FAK1 dependent manner^26,39,40^, and given the close relation of Simiate and FAK1 in dendritogenesis (Figs. 2, 3, 4, 5, 6, 7, 8, 9, 10), we wondered whether Simiate might be involved in NGF mediated dendritogenesis as well. Since our data suggests that the dissociation of nuclear Simiate and FAK1 between div 3 and div 5 is related to the onset of primary dendrite development, and since NGF is regulating transcriptional events underlying neuronal arborisation (reviewed in^41^), we speculated that NGF might affect nuclear FAK1 and Simiate. Thus, we evaluated the localisation of both proteins in div 3 primary hippocampal neurons after overnight application of NGF by performing costainings with several marker proteins for transcriptional activity and 3-dimensional reconstructions (Fig. 13 and Suppl. Table 1).

While we neither saw any effects on the volume of nuclear Simiate, nor on its colocalisation with FAK1, hnRNPs, newly synthesized RNA or Heterochromatin (Suppl. Table 1), nuclear FAK1 displayed an increase in its colocalisation with Heterochromatin and with sites of active transcription as visualized by DAPI staining and labelling of active RNAP2 (Fig. 13A,B). In order to analyse these findings in more detail, we measured the protein volumes. The data revealed that the proportions of active RNAP2, Heterochromatin and FAK1 as well as the proportions of FAK1 colocalised with active RNAP2 and Heterochromatin are significantly increased after NGF application (Fig. 13C), thus showing that nuclear FAK1 is involved in the regulation of gene expression triggered by NGF, but not nuclear Simiate.

Given the importance of dendritic growth cones in neuronal arborisation as well as their responsiveness to NGF on the one hand, and the dendrite promoting effects of FAK1 and Simiate (Figs. 8, 9, 10) on the other, we next investigated the presence of both proteins in dendritic growth cones after NGF application (Fig. 13D–F). While only little FAK1 and Simiate is seen in dendritic growth cones at div 3 without treatment (Fig. 13D), a striking increase in both proteins as well as in their colocalisation is observed following NGF exposure (Fig. 13E). Further analyses revealed an exceptional rise in the volume proportions of FAK1 and Simiate as well as in the proportion of proteins colocalised upon NGF treatment (Fig. 13F).

Taken together, the NGF experiments demonstrate that Simiate and FAK1 respond in a protein- and site-specific manner to the stimulation: while they cooperate in dendritic growth cones, in the nucleus, only FAK1 reacts to NGF by localising to Heterochromatin and sites of active transcription. Further, when looking at the changes in FAK1 and Simiate localisation during neuronal development (Figs. 2, 3, 4), the NGF experiments show that the de-colocalisation of nuclear Simiate and FAK1 from div 3 to div 5–7 is not a general feature of neurite outgrowth, but rather specific to primary dendrite development at this time.

## Discussion

Dendritogenesis is a complex cellular process, that is crucial to the correct functioning of neurons, but our understanding of the underlying molecular mechanisms is still fragmentary. Recent research resulted in a model, where interactions of transcription factors and the cytoskeleton regulate distinct facets and phases of dendritogenesis (reviewed in^1^). However, the proteins entailed as well as the molecular mechanisms engaged are not well understood yet.

### A model illustrating the interaction mechanisms of FAK1 and Simiate in dendritogenesis

We previously demonstrated that the protein Simiate binds to Actin and enhances dendrite formation upon elevated expression^13^, but the particular actions underlying the effect remained an open question. Here, we now show that Simiate is able to associate with FAK1 in the soma as well as in the nucleus, and that nuclear Simiate serves to specifically trigger primary dendrite development. By contrast, somatic Simiate enhances the branching of proximal dendrites and exaggerates dendrite growth, which, in turn, limits the extended transcription activity as well as the primary dendrite development induced by nuclear Simiate. FAK1, on the other hand, turned out to increase dendrite formation, branching and growth globally. In the nucleus, FAK1 and Simiate were shown to balance each other, thereby specifically renormalising the exaggerated primary dendrite development triggered by enhanced nuclear expression of Simiate.

**Figure 14:**
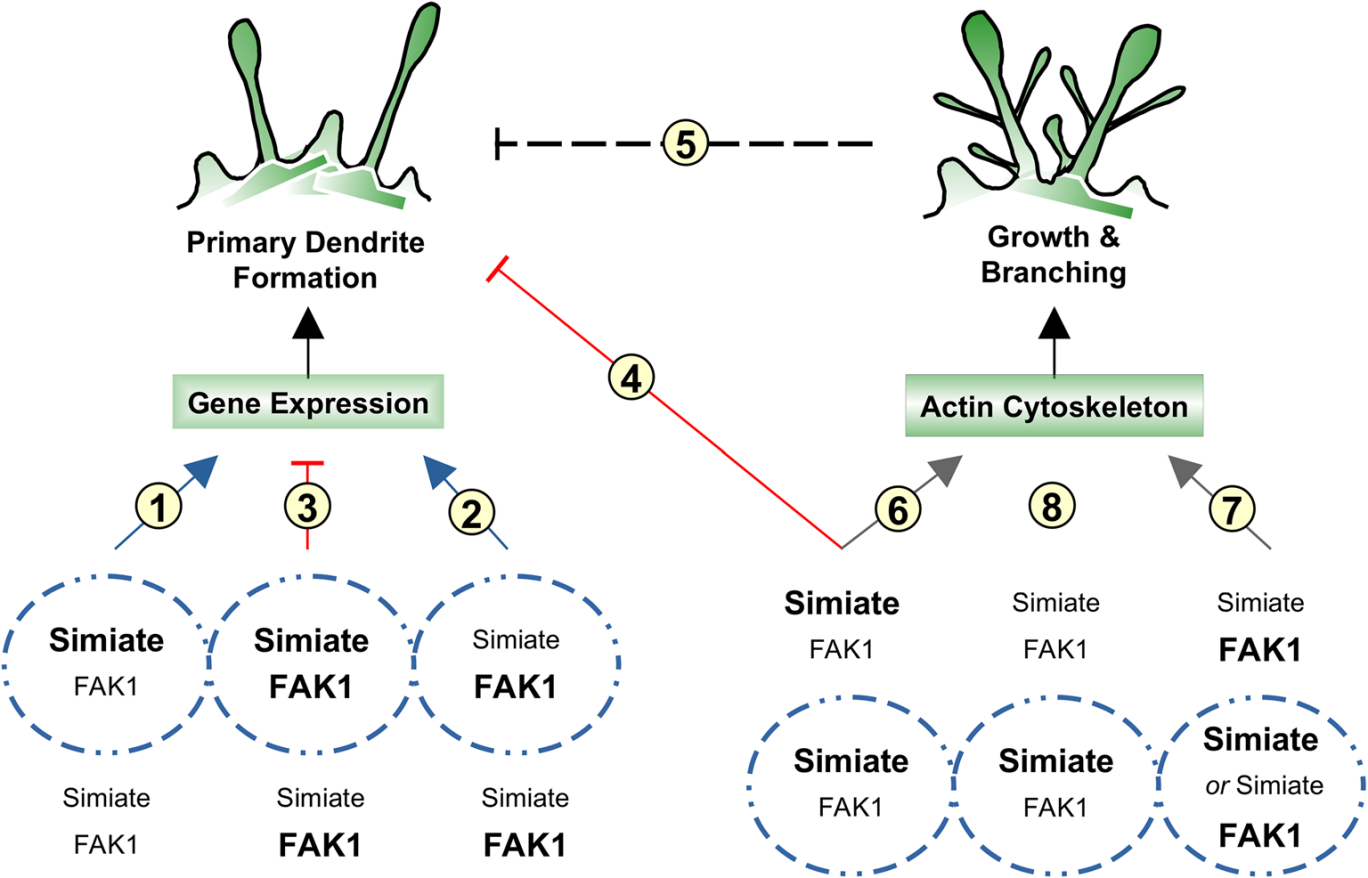
A model illustrating functions of FAK1 and Simiate in dendritogenesis based on our data. The picture depicts the putative molecular mechanisms underlying FAK1 and Simiate regulated dendritogenesis. Dotted circles represent nuclei, the surrounding area somata and dendrites. For both compartments, the expression levels of FAK1 and Simiate are indicated with either bold letters, representing enhanced expression, or regular letters, representing natural expression levels. (1) Nuclear Simiate influences gene expression and triggers primary dendrite formation. Based on our developmental data, this might occur around div 1, when unspecified protrusions are formed, and/or around div 5, an early phase of dendritogenesis: at both times, there is only little FAK1 available in the nucleus, and colocalisation of FAK1 and Simiate in the compartment is rare. (2) Enhanced FAK1 levels may also alter gene expression and increase dendritic complexity. (3) The specific renormalization observed for primary dendrites upon co-expression of FAK1 and Simiate implies that the two proteins are able to counterbalance each other, thereby holding primary dendrite formation. Based on our developmental data, this might occur around div 3, when there is an unusual high concentration of FAK1 in the nucleus and colocalisation with Simiate is also increased as well as past div 18, when mature expression levels are reached. (4) Somatic Simiate, which was found to preferentially induce dendritic growth and branching (cp. (6)), must be able to arrest primary dendrite formation as otherwise, primary dendrite formation would also be enhanced upon increased levels of somatic Simiate, which was not observed. The mechanisms underlying the finalization of primary dendrite formation are currently unknown though. (5) Such a mechanism could also be a signal originating from dendrite maturation (possibly caused by enhanced somatic Simiate expression), which goes back to the nucleus and finalizes primary dendrite formation via transcription regulation. However, it is also possible that somatic Simiate itself triggers a signalling cascade leading to the termination of primary dendrite outgrow. More research is required to evaluate these mechanisms. (6) In the soma, Simiate was found to preferentially cause dendrite growth and branching. (7) FAK1 induces branching and growth as well. Co-expression with nuclear Simiate does not alter the complexity of distal secondary and tertiary dendrites as compared to FAK1 expression alone, instead, the FAK1 phenotype persists. (8) Nuclear Simiate did not show any influence on higher order dendrites. (6–8) Based on the currently available data, it seems likely that somatic FAK1 and Simiate exert their functions in dendritogenesis by regulating Actin dynamics. The NGF experiment suggests that this may be the case in dendritic growth cones, where FAK1 and Simiate were found to colocalise along with Actin upon stimulation.

Figure 14 summarises our data in a model: while Simiate expression in somata induces dendritic growth and branching (Fig. 14.6), nuclear Simiate triggers primary dendrite development and enhances transcription as well as processing activity (Fig. 14.1, cp 43). FAK1 is also able to enhance transcription and to trigger primary dendrite formation (Fig. 14.2), but a co-expression of both proteins results in a renormalisation of primary dendrite complexity (Fig. 14.3). Based on our developmental data, such FAK1-Simiate mediated augmentations of primary dendrite formation might occur around div 1, when unspecified protrusions are formed, and/or around div 5, an early phase of dendritogenesis, but rather not around div 3 as a co-expression of both proteins prevented the enhanced primary dendrite formation typical to nuclear Simiate (Fig. 14-1&2).

Since there is no effective signal known, that could be used to generate a somatic protein localisation, the construct employed for Simiate expression in somata is also seen in the nucleus, but the absence of increased primary dendrite formation, which is characteristic to nuclear Simiate expression, suggests that either somatic Simiate or the branching itself trigger a signal terminating primary dendrite formation initiated by nuclear Simiate (Fig. 144&5). In line with this finding, the increased transcription and processing activity observed during nuclear Simiate expression is not seen during ubiquitous expression of the protein. Remarkably though, FAK1, although likewise present in the soma and the nucleus, does not have such an endpoint effect, but instead induces both, primary dendrite formation as well as dendritic branching (Fig. 14-2&6), thus showing that the observed termination effect is specific to somatic Simiate or its particular role in dendrite development. Indeed, the increase in endogenous Simiate between div 10 and div 14, when primary dendrite formation is being completed (cp.^42,43^), parallels the experiment.

It is noteworthy that the dendrite arresting behaviour of somatic Simiate strikingly reassembles the ability of Snapin to enhance dendritic branching while decreasing primary dendrite numbers at the same time^42^. Furthermore, increased expression of the postsynaptic density protein PSD95, which is well-known for its role in synapse maturation, was recently shown to stop the outgrowth of secondary dendrites independent of any neuronal activity^44,45^. Taken together, these results suggest that the intrinsic regulation controlling arborisation in primary hippocampal neurons engages top-down mechanisms to coordinate the different phases of dendrite development: proteins functioning in the establishment of structures characteristic to a higher level of maturation (e.g. secondary dendrites or synapses) are able to directly or indirectly end the growth of structures characteristic to the preceding maturation stage (e.g. primary dendrite formation or branching). These molecular mechanisms act independently from actual neuronal activity or other extrinsic factors. The idea is in line with the choreography-like development of hippocampal neurons in cultures^35^. However, though reports on factors increasing dendritic complexity are numerous, more information on the mechanisms coordinating and confining distinct phases of arborisation is currently missing.

### Nuclear functions of FAK1 and simiate

Both proteins, FAK1 and Simiate, are present in the nucleus. Although FAK1 has recently been found to regulate gene expression (reviewed in^16,19^), the functions of nuclear FAK1 are not well understood yet, in particular not in neurons. Interestingly, studies in muscle cells showed that nuclear FAK1 serves to promote cell differentiation via Chromatin remodelling^17,21^, while experiments performed in neurons illustrated FAK1 to exaggerate dendritogenesis^26^, thus suggesting that neuronal FAK1 could control dendrite formation by regulating gene expression.

Our experiments indeed support this idea: not only have we found a significant increase in dendritic complexity upon enhanced FAK1 expression, but also increased transcription activity and massively elevated levels of newly synthesized RNA. Since these effects were not accompanied by a significant colocalisation of FAK1 and corresponding marker molecules, it seems likely that the examined complexes are not directly targeted by FAK1. By contrast, NGF triggered neurite outgrowth, which requires FAK1^26,39,40^, resulted not only in increased transcription activity, but also in significantly increased proportions of FAK1 colocalising with Heterochromatin and with sites of active transcription. These findings show that FAK1 is indeed able to target gene expression in the context of dendritogenesis, and that the specific actions of nuclear FAK1 depend on the respective cellular context.

In addition, we found that FAK1 associates with Simiate, which itself is also able to trigger transcription activity. Remarkably though, the association of nuclear Simiate and FAK1 turned out not to further increase transcription and dendrite complexity, but to limit primary dendrite formation, as evinced by co-expression studies as well as a characteristic de-colocalisation of endogenous FAK1 and Simiate in the nucleus around div 5–7, the time after onset of primary dendrite formation.

Inside the nucleus, Simiate prefers to associate with FAK80, a Calpain2 cleavage product, which contains the FERM and the kinase domain of FAK1^22,23^, and which translocates into the nucleus upon neurite outgrowth^46^. Nuclear functions of the kinase domain are unknown, but the FERM domain has been shown to serve as a scaffold promoting ubiquitination and degradation of transcription factors via the 26S proteasomal pathway^47^. The cellular relevance is not yet clear though. While FAK1 mediated degradation of transcription factors has been speculated to either enhance turnover^19^ or to reduce gene expression^15^, the protein has also been proposed to enable the expression of specific genes, such as components and regulators of cytoskeleton dynamics or synapse kinetics^16^. Moreover, the association of p53 with the FERM domain has been found to inhibit the transcriptional activity of p53^48^. These studies thus suggest that the association of FAK1 and Simiate in the nucleus might serve to regulate the activity of Simiate. Indeed, our data support an inhibitory role for the interaction: while nuclear Simiate expression massively increases transcription activity, a co-expression with FAK1 reduces not only active transcription and increases inactive chromatin, but also renormalises the exaggerated primary dendrite formation typical to enhanced expression of nuclear Simiate. On the molecular level, these results are supported by significant levels of GFP-FAK1 in the nucleus and a striking attraction of FAK1 by nuclear Simiate.

More research is needed to identify the genes targeted by FAK1 and Simiate, and to evaluate the underlying molecular mechanisms in detail.

### FAK1 and Simiate in dendrites

In dendrites, a colocalisation of FAK1 and Simiate is usually rare (cp. Fig. 2A,B), although overall protein levels are comparable. Nonetheless, a significant increase in the colocalisation of both proteins is seen from div 15 to div 18, when dendrites mature and synapses develop, implying a role for the interaction of both proteins in dendritic growth and branching. Such a developmental role of FAK1 and Simiate in dendrites is supported by the Sholl analyses (cp. Fig. 9F,G) as well as by the profound colocalisation of both proteins observed in dendritic puncta upon recombinant FAK1 expression (Fig. 7B). Since endogenously, Simiate is absent from synapses (Derlig 1. Paper) as well as from most FAK1-positive puncta, the character of these sites is currently unclear though.

Noteably, neurotrophin stimulation induced a significant increase in the colocalisation of FAK1 and Simiate at a well-known dendritic site, the dendritic growth cone^13,26^. Dendritic growth cones are different to axonal growth cones not only in moving speed, but also in microtubule dynamics as well as in lamelli- and filopodia behaviour^49,50^, however, the precise relationship between cytoskeleton organisation in dendritic growth cones and neuronal arborisation remains poorly characterised. Recent investigations revealed that even minor changes in the Actin dynamics of dendritic growth cones are sufficient to significantly alter dendrite growth and branching during development^50,51^. Given the role of FAK1 in Actin polymerisation (reviewed in^15^), Simiate's direct association with Actin^13^, and the colocalisation of both proteins with Actin in growth cones during dendritogenesis^13,52^, it is plausible that the effects of somatic FAK1 and Simiate on dendrite length and branching involve a FAK1-Simiate mediated regulation of Actin dynamics in dendritic growth cones. The idea is supported by the fact that dendrite length and branching are neither controlled by nuclear Simiate, nor by a balanced expression of FAK1 and Simiate in the nucleus, but instead significantly exaggerated by enhanced expression of FAK1 or Simiate in the soma. Indeed, our analyses also demonstrated increased levels of FAK1 and Simiate colocalising in Actin-rich areas of dendritic growth cones after NGF treatment. Both data are in line with the fact that NGF has not only been shown to rearrange the Actin cytoskeleton in dendritic growth cones^53,54^, but to also decrease the number of primary dendrites and to increase the dendrite length^55^ as observed for enhanced somatic expression of Simiate.

## Supplementary Information

Supplementary Information 1.

Supplementary Information 2.

Supplementary Information 3.

## Data Availability

The datasets used and/or analysed during the current study are available from the corresponding author on reasonable request.
