# Supplementary material for: Simiate and the focal adhesion kinase FAK1 cooperate in the regulation of dendritogenesis
Source: Sci Rep. 2022 Jul 4;12:11274. doi: 10.1038/s41598-022-14460-y (PMC9253104; doi:10.1038/s41598-022-14460-y)
Supplement: Supplementary file 1 — Supplementary Information 1. [file 41598_2022_14460_MOESM1_ESM.pdf]

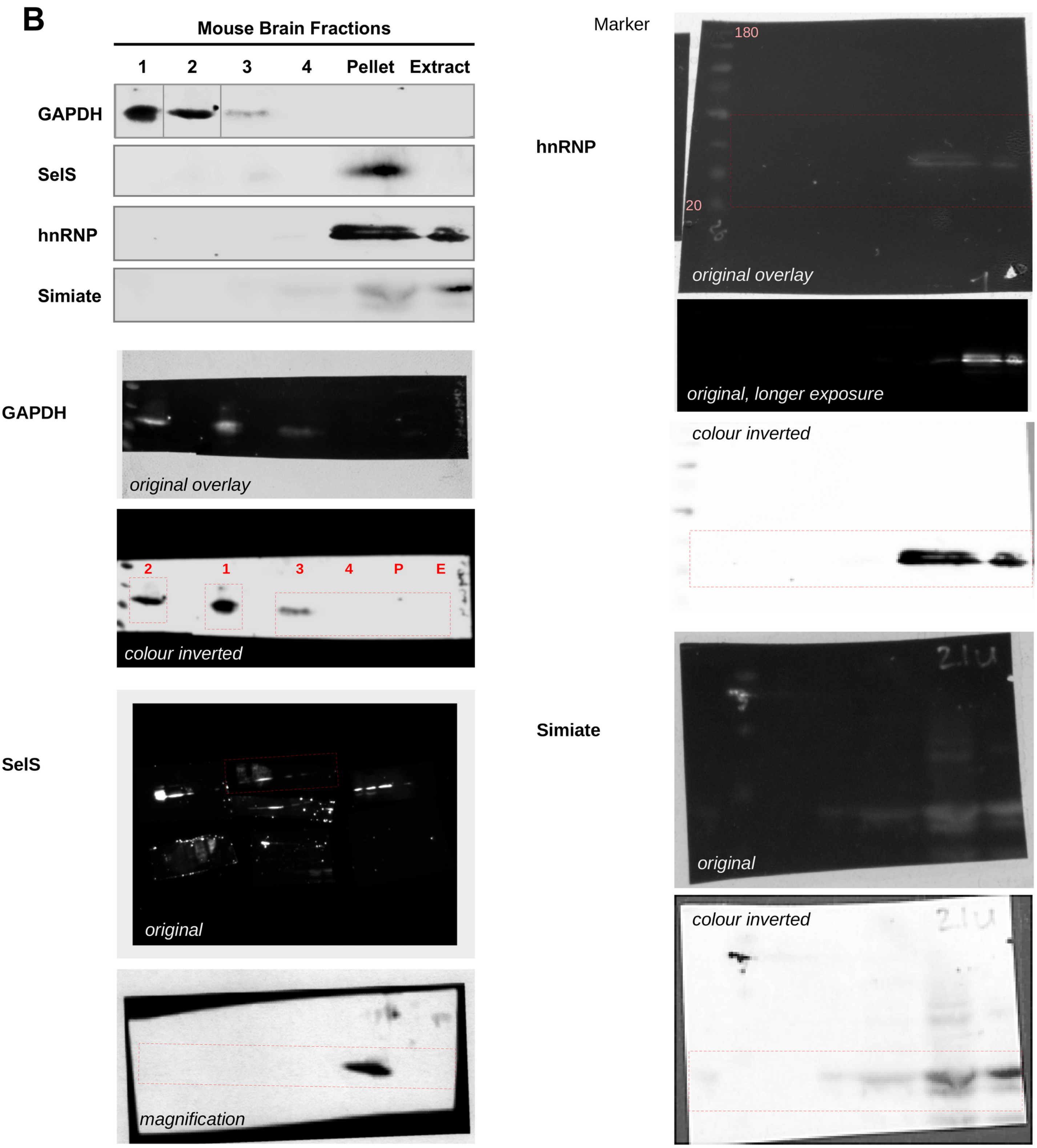

Supplement for Figure 1B: FAK1 and Simiate associate (B). B) Nuclear fractioning. 1 - Mouse brain homogenate, 2 - Mouse brain cytosol, 3 - Pellet 500rcf, 4 - Supernatant 70000rcf, Pellet – final pellet containing nuclear proteins not extracted, Extract - proteins successfully extracted from cell nuclei. Please kindly note that for reasons of animal care (reduction of animals used in experimentation), some blots were cut according to the expected protein seizes prior to antibody application in order to save material. All images were taken using the Bio-Rad Gel Doc system and stained as indicated on the left. Western Blots are shown as overlay images generated from colorimetric pictures and pictures of the ECL signal with grey tone bands on a light background. Boxes indicate the bands shown in Figure 1B. To improve the comparability, grey tones were adjusted for the figure. In the case of GAPDH, due to a different loading order, the lanes were rearranged to fit the figure. Empty spaces in between result from lanes carrying only loading buffer in order to fill all pockets of the gel. The Western Blot for SelS was performed along with several other blots, which are also present on the picture. Please note that the image was mirrored due to back-to-front blotting and magnified as shown in the following picture "magnification". Since in the case of Simiate the last proteins already touch the rim of the nitrocellulose membrane, the colour inverted picture was contrasted to distinguish band and background. P: Pellet, E: Extract.

C

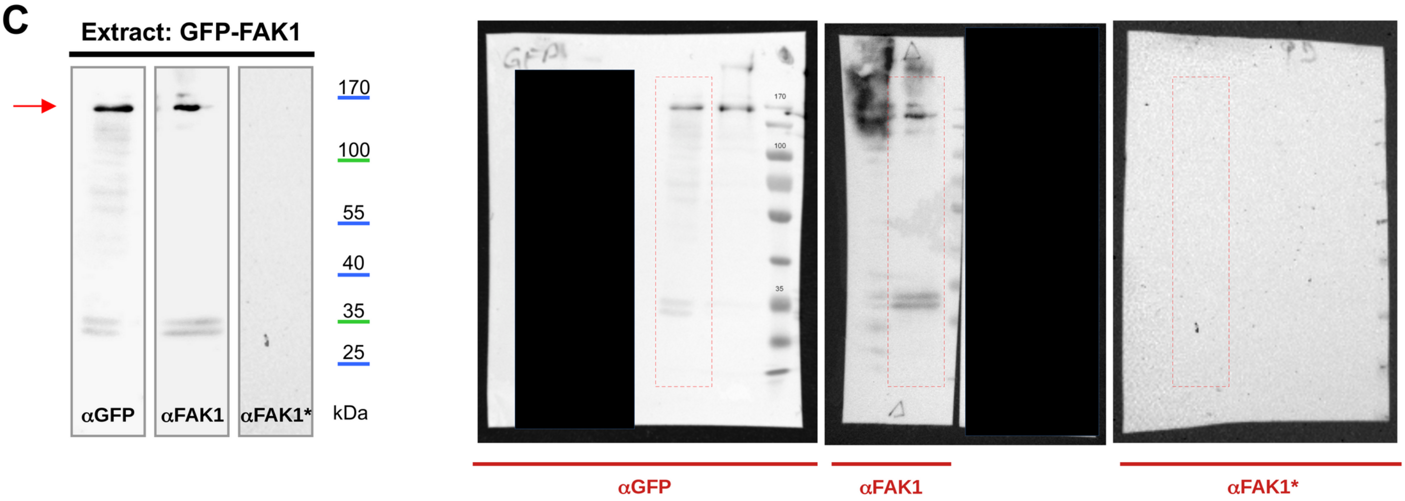

Supplement for Figure 1C: C) Nuclear GFP-FAK1 is specifically detected by αFAK1. The Western blot stripes show extracts from nuclei of GFP-FAK1 expressing HEK293 cells stained αGFP, αFAK1 and αFAK1 preincubated with GFP-FAK1 (αFAK1*). Please kindly note that in order to save membrane material, blots were cut according to the lanes required prior to antibody hybridisation. The lanes shown in Figure 1C are highlighted by red boxes. All images are colour inverted overlays obtained with the Bio-Rad Gel Doc system and stained as indicated below. Black boxes cover parts of the gels that are not involved in the experiment to comply with copy right regulations.

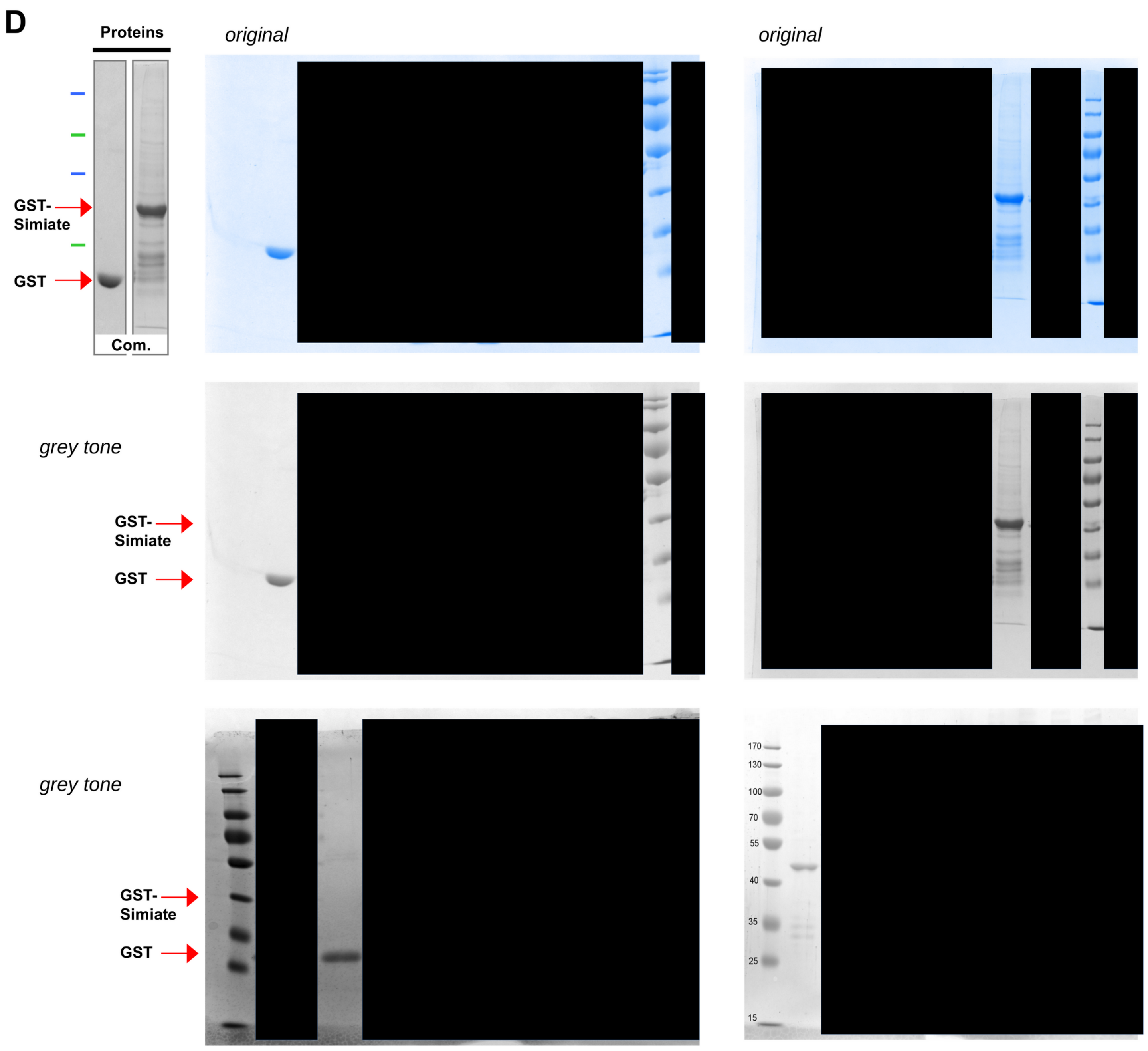

Supplement for Figure 1D: D) A Coomassie-staining visualises the proteins used as baits in the subsequent coprecipitation experiments. To improve the visibility of details, original Coomassie stained gels (original) were changed to grey tones. Please note that the imaged area depends on the zoom used for taking the picture. Parts of the gels that are not related to the experiment are covered with black boxes in order to comply with copy right regulations. Bottom Panel: An additional example of a bait preparation illustrates the consistent quality of the recombinant proteins manufactured.

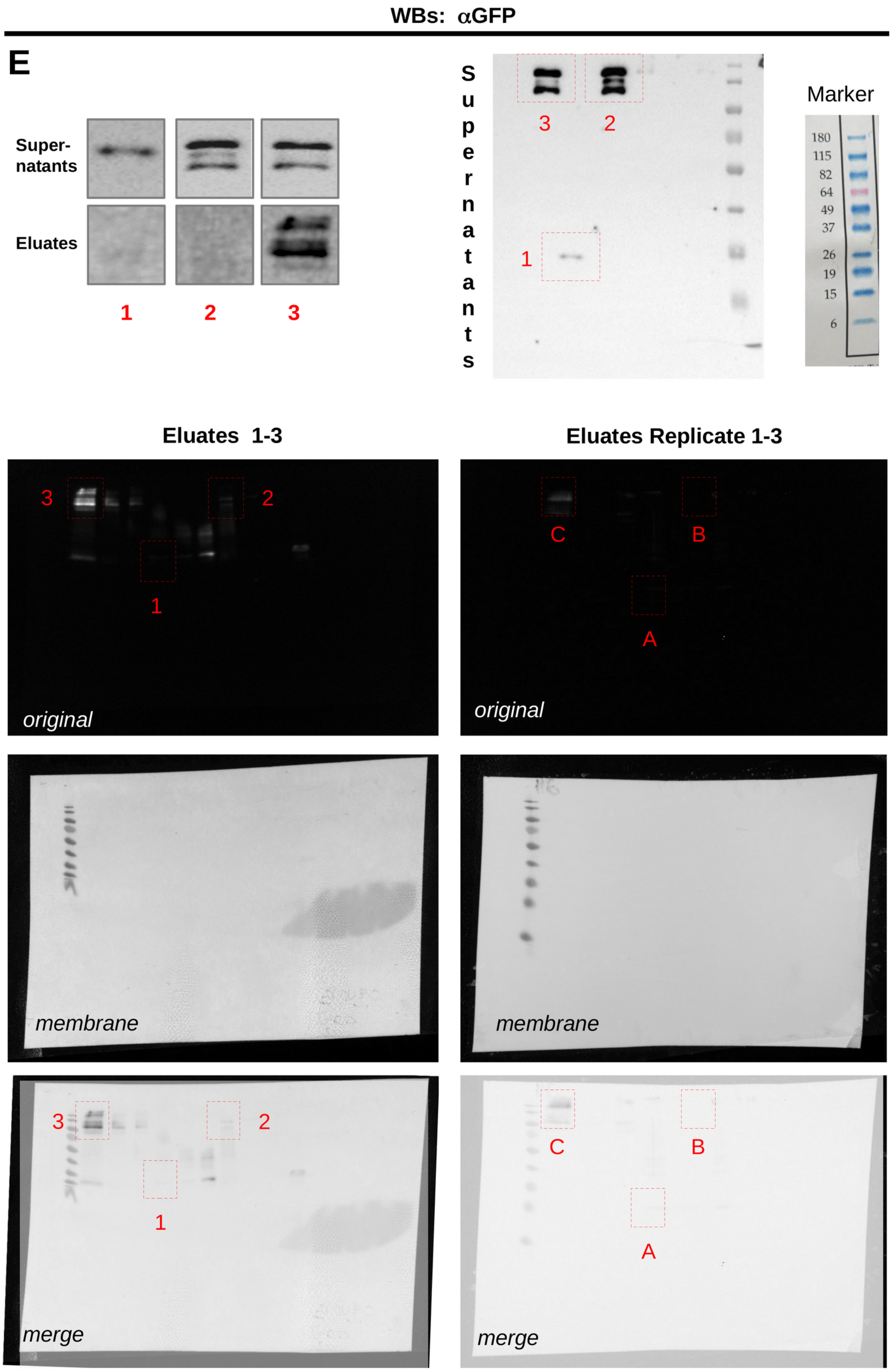

Supplement for Figure 1 E: FAK1 and Simiate associate.

E) Cytosolic GFP-FAK1 binds to GST-Simiate. The images show Figure 1E and the original blots as well as replicates. Please note that the original bands have been reassembled and colour inverted to fit the figure. Corresponding bands were therefore indicated by boxes.
The panels below illustrate the composition of Western Blot pictures: shown are the original pictures taken from ECL signal on the respective Western Blots (black background, light bands, "original"), colorimetric images demonstrating the prestained marker (light background, grey bands, "membrane") and overlays (light background and grey bands, "merge") as imaged with Bio-Rad Gel Doc. Usually, only merged pictures are shown as they contain all information. The blots were stained for GFP.

Labelling:
In all pictures, the labelling is as follows: 1 and A: GST-Simiate + GFP; 2 and B: GST + GFP-FAK; 3 and C: GST-Simiate + GFP-FAK1; merge: membrane with prestained marker and ECL signal as shown above, membrane: membrane with prestained marker only.

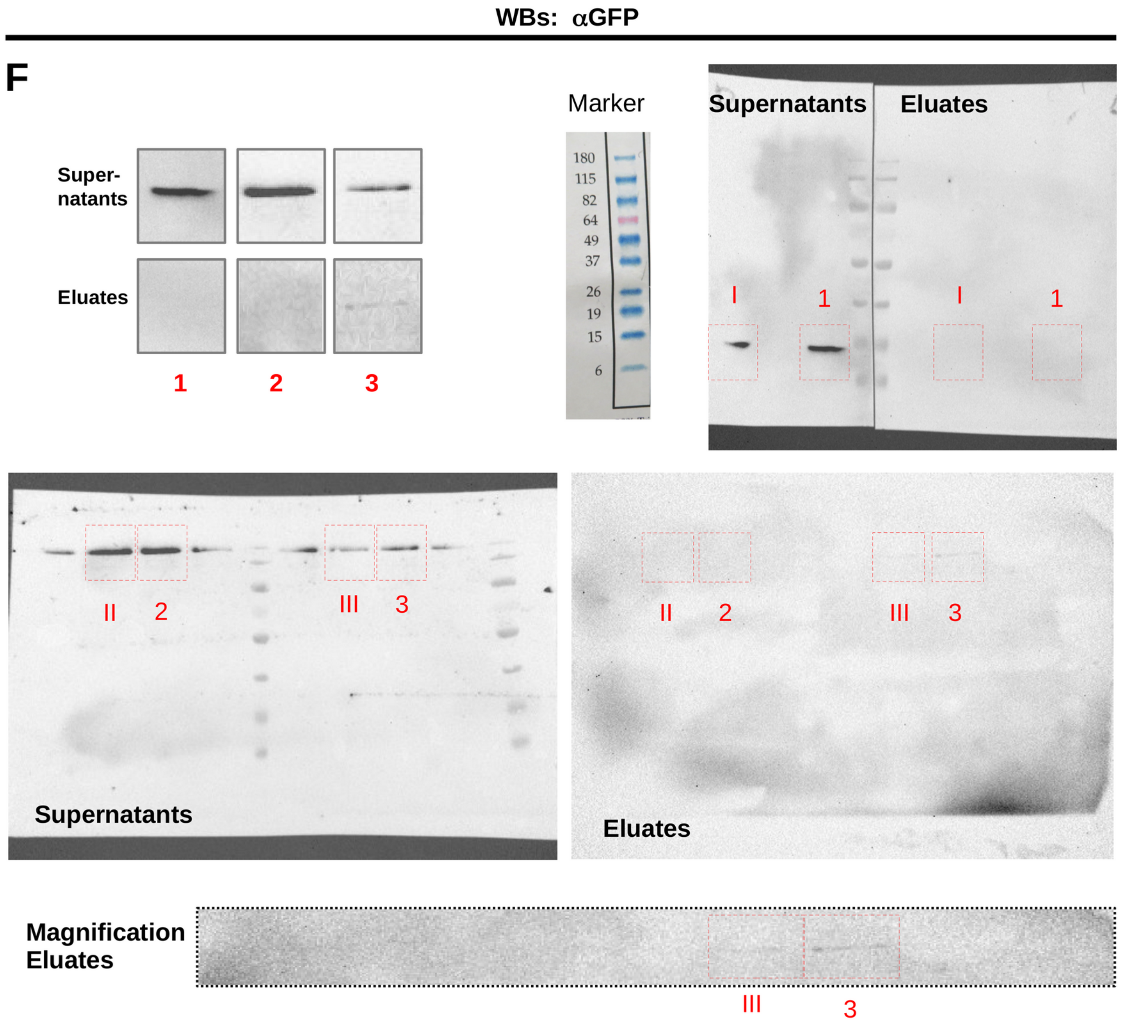

Supplement for Figure 1F: FAK1 and Simiate associate. F) Nuclear GFP-FAK1 binds to GST-Simiate. The images show Figure 1F and related original blots. Please note that the original bands have been reassembled to fit the figure. Corresponding bands were therefore indicated by boxes.

All images are overlays compiled from colorimetric pictures of the Western Blot membranes and the ECL signal as imaged with Bio-Rad Gel Doc. The blots were stained for GFP.

<u>Labelling</u>:

1, I: GST-Simiate + GFP; 2, II: GST + GFP-FAK; 3, III: GST-Simiate + GFP-FAK1. Arabic numbers: RIPA buffer with 0.5% Triton, roman numbers: RIPA buffer with 300mM NaCl. For more details, please refer to the methods section.

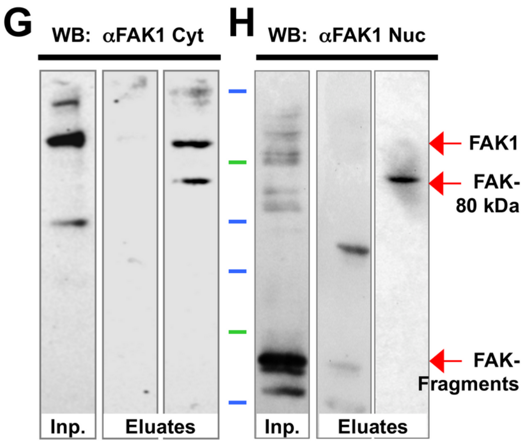

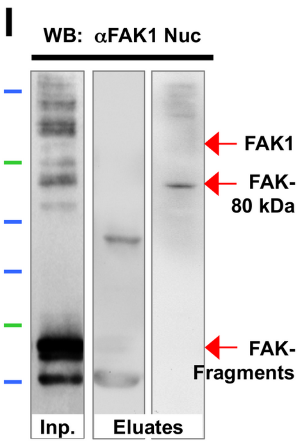

I) Supplement for Figure 1H:
Inside the nucleus, the FAK80-Simiate interaction is stable at different buffer conditions, while the association with full length isoforms is rather weak and variable, however, also detectable. The Western blot stripes show extracts from nuclei purified from brains of adult mice. Both experiments were performed with different RIPA buffers: H - standard RIPA buffer with additional 100 mM NaCl and 0.5 % Triton; I - standard RIPA buffer with additional 100 mM NaCl.

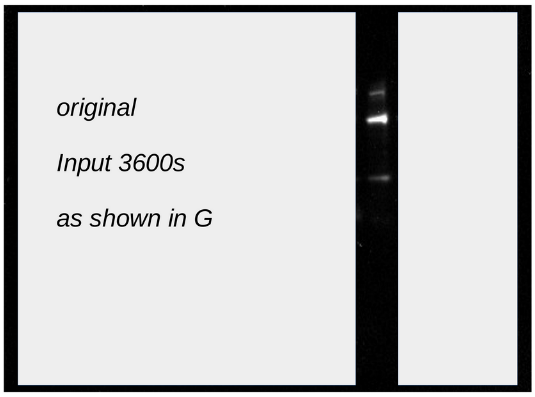

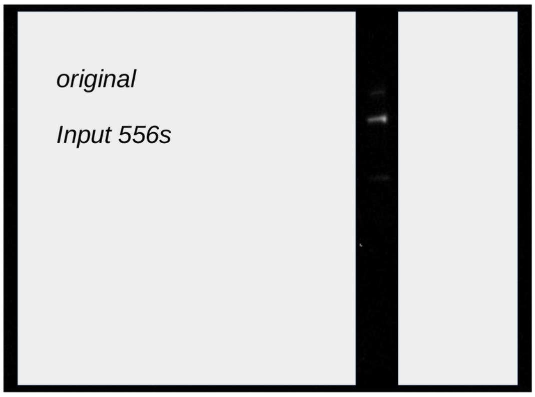

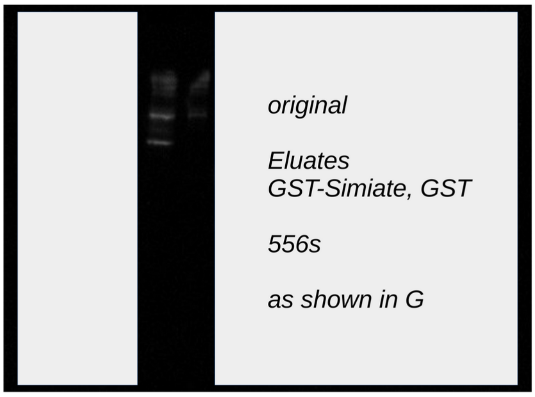

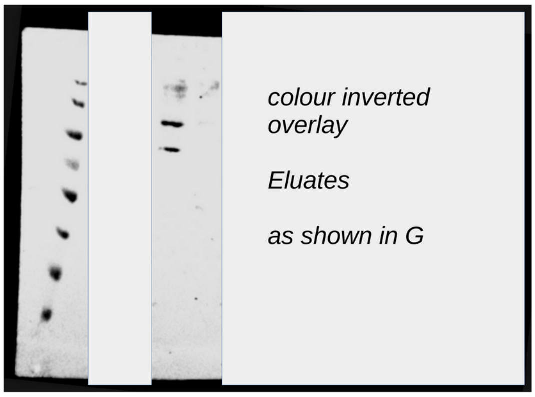

original (merge)

green box: *Input for H* — *blue box:* *Input for I*

yellow box: *Input for a replicate of G*

original

green boxes: *Eluates for H* — *blue boxes:* *Eluates for I*

yellow boxes: *Eluates of a replicate of G*

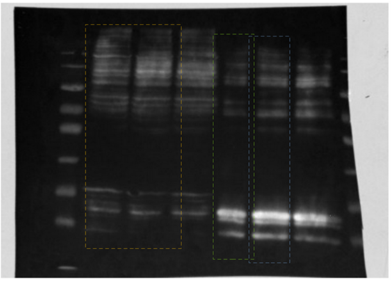

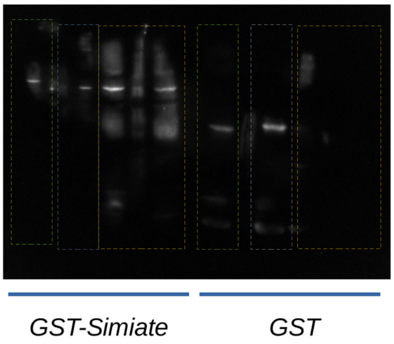

Supplement for Figure 1G,H

G) Simiate interacts with endogenous FAK1 and FAK80, a FAK1 isoform generated by Calpain2 cleavage (cp. A).
H) In the nucleus, Simiate prefers FAK80.

Both experiments (G,H) show a significant enrichment of FAK80 in the precipitate by Simiate, even from below detection levels (G), suggesting a strong association of both proteins. All proteins were extracted from adult mouse brains. To improve the visibility of details, original Coomassie stained gels (original) were changed to grey tones. Please note that the imaged area depends on the zoom used for taking the picture. Parts of the gels that are not related to the experiment are covered with grey boxes in order to comply with copy right regulations.

Bottom Panel on the left: An additional example of a bait preparation illustrates the consistent quality of the recombinant proteins manufactured. Right Panels: Blue boxes indicate the lanes shown in Figure 1H, green boxes a repetition of the experiment under different buffer conditions, and yellow boxes show a repetition of G with two other buffers.

The additional bands observed for GST at around 30 kDa could be caused by unspecific binding of FAK1 fragments due to the high amount present in nuclear fractions and/or by an unspecific interaction of antibodies with GST itself. The nature of the additional band at app. 40 kDa is unclear though, however, no bands of any of these sizes are seen for GST-Simiate, hence supporting a specific interaction of FAK1 and Simiate.
