# Supplementary material for: Simiate and the focal adhesion kinase FAK1 cooperate in the regulation of dendritogenesis
Source: Sci Rep. 2022 Jul 4;12:11274. doi: 10.1038/s41598-022-14460-y (PMC9253104; doi:10.1038/s41598-022-14460-y)
Supplement: Supplementary file 3 — Supplementary Information 3. [file 41598_2022_14460_MOESM3_ESM.pdf]

Supplemental Figure for Figure 9

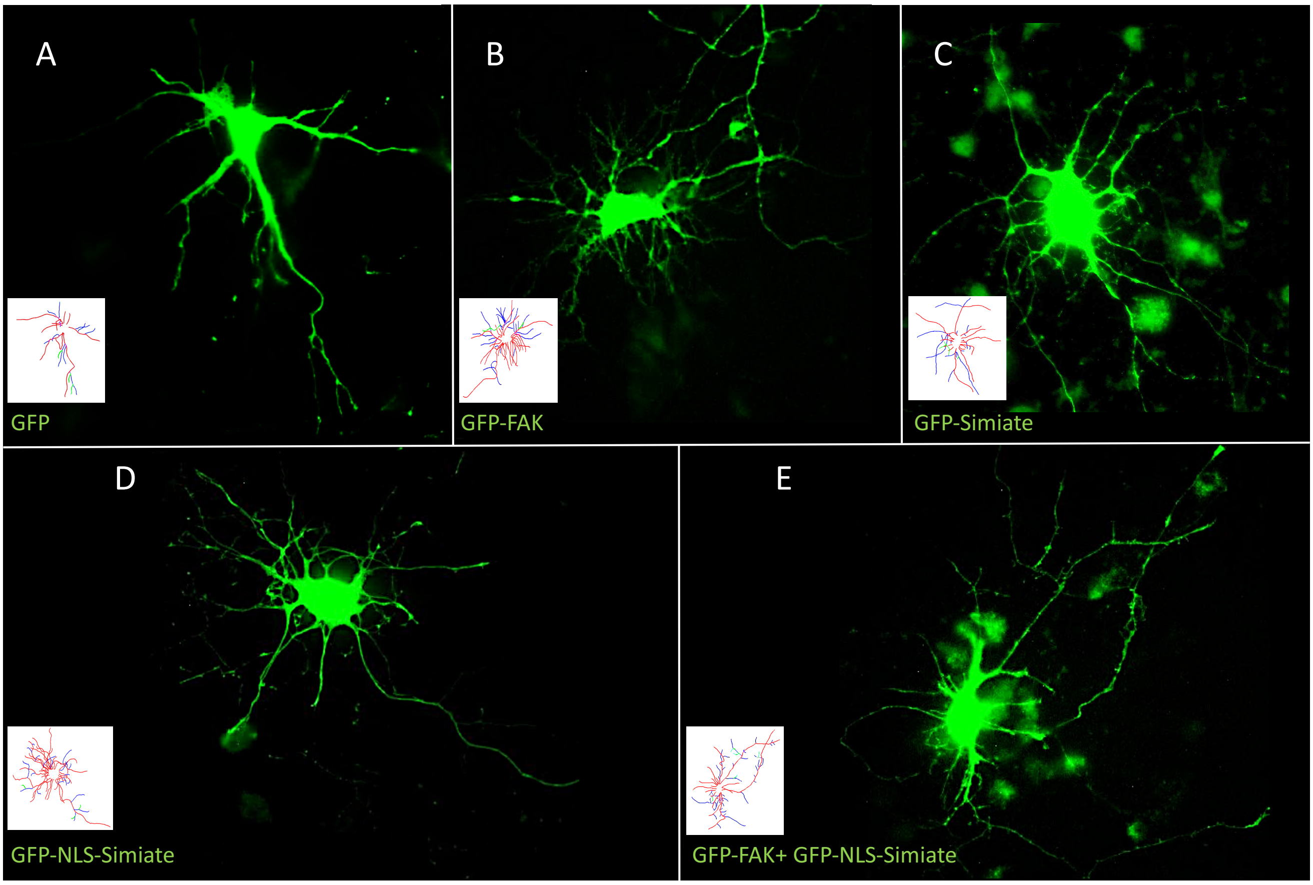

Supplemental Figure for Figure 9: FAK1 and Simiate have different effects on primary, secondary and tertiary dendrites. In order to analyse the dendritic complexity of neurons, div 8 neurons were imaged and dendrites were tracked based on GFP signal and MAP2 stainings using NeuronJ, a plug-in for Fiji software. A-E) The pictures show div 8 primary hippocampal neurons following over-night expression of the indicated constructs.
